# Exercise enhances motor skill learning by neurotransmitter switching in the adult midbrain

**DOI:** 10.1038/s41467-020-16053-7

**Published:** 2020-05-04

**Authors:** Hui-quan Li, Nicholas C. Spitzer

**Affiliations:** 1 Neurobiology Section, Division of Biological Sciences and Center for Neural Circuits and Behavior, La Jolla, CA 92093-0357 USA; 2 Kavli Institute for Brain and Mind, University of California San Diego, La Jolla, CA 92093-0357 USA

**Keywords:** Motor control, Inhibition-excitation balance, Neurotransmitters

## Abstract

Physical exercise promotes motor skill learning in normal individuals and those with neurological disorders but its mechanism of action is unclear. We find that one week of voluntary wheel running enhances the acquisition of motor skills in normal adult mice. One week of running also induces switching from ACh to GABA expression in neurons in the caudal pedunculopontine nucleus (cPPN). Consistent with regulation of motor skills, we show that the switching neurons make projections to the substantia nigra (SN), ventral tegmental area (VTA) and ventrolateral-ventromedial nuclei of the thalamus (VL-VM). Use of viral vectors to override transmitter switching blocks the beneficial effect of running on motor skill learning. We suggest that neurotransmitter switching provides the basis by which sustained running benefits motor skill learning, presenting a target for clinical treatment of movement disorders.

## Abstract (web summary)

Exercise promotes motor skill learning via unclear mechanisms. Here, the authors show that running wheel training results in neurotransmitter switching in caudal pedunculopontine nucleus neurons of mice. These neurons project to several brain regions, regulating the acquisition of motor skills.

## Introduction

Motor skill learning is fundamental to everyday life and is regulated by a neural network involving the cortex, thalamus, basal ganglia, brain stem, cerebellum, and spinal cord^1,2^. Both neuronal and glial plasticity are essential for motor skill learning and disruption of this plasticity causes motor deficits^3,4^. Aerobic physical exercise promotes the ability to acquire new motor skills^5^ and serves as a therapy for many motor disorders^6–8^, but its basis of action is not well understood. Running is a natural motor activity for mice^9^ and generates plasticity in multiple brain regions^4,10,11^. Neurotransmitter switching is a newly appreciated form of plasticity that refers to the ability of neurons to change their transmitter identity in response to sustained stimuli, typically leading to changes in behavior^12^. We hypothesized that chronic running induces neurotransmitter switching in a circuit that is important for motor skill learning.

We find that mice that have run on a wheel for a week have an enhanced ability to acquire motor skill on the rotarod and balance beam. This enhancement in learning is temporally correlated with an activity-dependent transmitter switch of midbrain cholinergic neurons that now express GABA. The change in transmitters corresponds to changes in the levels of transcripts of their transmitter synthetic enzymes. The switching neurons project to nuclei that regulate motor skill learning, including the SN, VTA, and VL-VM. Overriding the switch using viral tools to restore the loss of the synthetic enzyme for ACh or prevent the gain of the synthetic enzyme for GABA blocks enhancement of the ability to acquire motor skills.

## Results

### Sustained running enhances motor skill learning

**Fig. 1:**
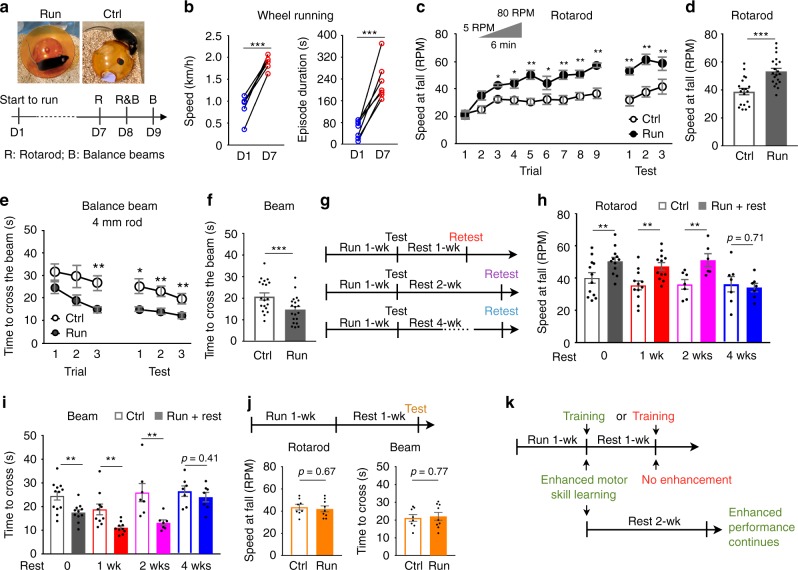
Sustained running enhances motor skill learning. **a** Top: Runner mice were housed with running wheels and control mice with wheelbases. Bottom: timeline for running and behavioral tests. **b** Mean speed (left, *n* = 6 animals per group) and mean episode duration (right, *n* = 7 animals per group) of running on day 1 (naive) or day 7 (1-week trained). **c** Speed at fall on an accelerative rotarod of each trial during training or each test the day after training. **d** Mean speed at fall on this rotarod in three tests on the day after training. **e** Time to cross a 1-m long, 4-mm diameter rod balance beam during each trial of training or each test the day after training. **f** Mean time to cross this balance beam in three tests the day after training. For **c**–**f**, *n* = 19 Ctrls and 20 Runners. **g** Timelines for immediate behavioral testing and retesting. **h**, **i** Mice that had run for 1 week or non-runner controls were tested with (**h**) rotarod and (**i**) balance beam (4 mm rod). After rest for 1, 2 or 4 weeks, mice were retested. For **h**, *n* = 12 animals per group for 0 and 1 week, 7 Ctrls and 6 Runners for 2 weeks, and 7 per group for 4 weeks. For **i**, *n* = 12 animals per group for 0 week, 10 for each group for 1 week, 7 per each group for 2 and 4 weeks. **j** Top: timelines for delayed behavioral testing. Bottom: performances on rotarod (left) and balance beam (right, 4 mm rod) after 1 week of running followed by 1 week of rest compared to controls that never ran. *n* = 8 animals for Ctrl and 9 for Run+Rest. **k** Time dependence of enhanced motor skill learning. When motor skills are trained (green) immediately after 1 week of running, runner mice show enhanced learning. Enhancement of acquired motor skills is sustained for at least two weeks. When motor skills are trained (red) after 1 week of running followed by 1 week of rest, no enhancement in learning is observed. Statistical significance *p < 0.05, **p < 0.01, ***p < 0.001 was assessed by two-sided paired *t*-test (**b**) or two-sided Welch’s *t*-test (other panels). Data shown are mean ± SEM.

We investigated the impact of chronic voluntary running on motor skill learning by exposing adult mice to running wheels for one week (Fig. 1a). Each mouse spent consistent time on the wheel every day, indicating continuous interest, and their running skill improved as measured by increased running speed, increased duration of running episodes and more stable movement on the wheel (Fig. 1b and Supplementary Fig. 1a, b). At the end of the running period we assessed performance on the accelerating rotarod and narrow balance beam to evaluate motor skill acquisition^13–15^. In comparison to mice without running wheels, mice that ran for one week demonstrated enhanced learning of motor skills, mastering an accelerating rotarod more rapidly and accommodating to balance beams more quickly (Fig. 1c–f and Supplementary Fig. 1c, d).

We calculated the slopes of learning curves to assess the speed of motor skill learning. Mean data points for each training trial were plotted and fitted and the coefficient of determination (*R*^2^) was used to justify the fit. The mean speed at fall from a rotarod for the nine trials on the training day was fitted by a one-phase association model (*R*^2^ > 0.96 for controls; *R*^2^ > 0.95 for runners).

1 $$y(t) = y_0 + (A - y_0)\left( {1 - {\rm{e}}^{ - kt}} \right)$$

*y*(*t*) is the speed at fall on trial number *t*, *y*_0_ is the speed at fall for the first trial (*t* = 0), *A* is the plateau and *k* is a rate constant.The slopes were calculated with

2 $$y\prime ( t ) = \frac{{{\rm{d}}y}}{{{\rm{d}}t}} = k{\rm{e}}^{ - kt}(A - y_0)$$

To calculate the initial slope, *y*′(0), we used

3 $$y\prime ( 0 ) = g\left( {k,A} \right) = k(A - y_0)$$

The standard errors were calculated with

4 $$\frac{{\partial g}}{{\partial k}} = (A - y_0)$$

5 $$\frac{{\partial g}}{{\partial A}} = k$$

6 $$\delta g = \sqrt {\left( {\frac{{\partial g}}{{\partial k}}\delta k} \right)^2 + \left( {\frac{{\partial g}}{{\partial A}}\delta A} \right)^2} = \sqrt {\left( {A - y_0} \right)^2\delta k^2 + k^2\delta A^2}$$

The values of the slopes are presented as *g* ± *δg* (mean ± sem). Initial slopes were 17 ± 4 rpm/trial for runners and 5 ± 2 rpm/trial for controls, significantly steeper for the runners (*p* = 0.01, Welch’s *t*-test) (Fig. 1c).

The mean time to cross a 4 mm rod beam for the three trials on the training day was fitted by linear regression (*R*^2^ > 0.98 for controls; *R*^2^ > 0.99 for runners)

7 $$y( t ) = y_0 + kt$$

*y*(*t*) is the time to cross the beam on trial number *t*, *y*_0_ is the time for the first trial (*t* = 0) and *k* is the slope

8 $$y\prime ( t ) = k$$

The values of the slopes are presented as *k* ± *δk* (mean ± sem). The slopes were −4.7 ± 0.5 s/trial for runners and −2.4 ± 0.3 s/trial for controls, again significantly steeper for runners (*p* < 0.001, Welch’s *t*-test) (Fig. 1e). Additionally, sustained running improved rotorod and balance beam test performance (Fig. 1d, f and Supplementary Fig. 1d) but did not affect basal locomotor activity as measured by infrared beam crossings in home cages (Supplementary Fig. 1e, f). The steeper slopes of the behavioral acquisition curves demonstrate enhanced learning relative to the controls, in accordance with previous studies^16,17^. If the change in behavior induced by wheel running were one of performance, these curves would be parallel and the slopes would be equal.

To explore further the effect of running on enhancement of motor skill learning, we removed running wheels from mouse cages after the first motor skill tests and retested the mice after different resting periods (Fig. 1g). Enhanced performance on both rotarod and balance beam was sustained up to 2 weeks but not 4 weeks (Fig. 1h,i). Moreover, when mice were not trained on the rotarod and balance beam until 1 week after running, their motor skill learning was not enhanced (Fig. 1j). This result suggests that running creates a sensitive period in the adult brain during which motor skill learning is improved and shows that the gain in motor skills persists longer than the duration of this period (Fig. 1k).

### Running induces transmitter switching in the midbrain cPPN

**Fig. 2:**
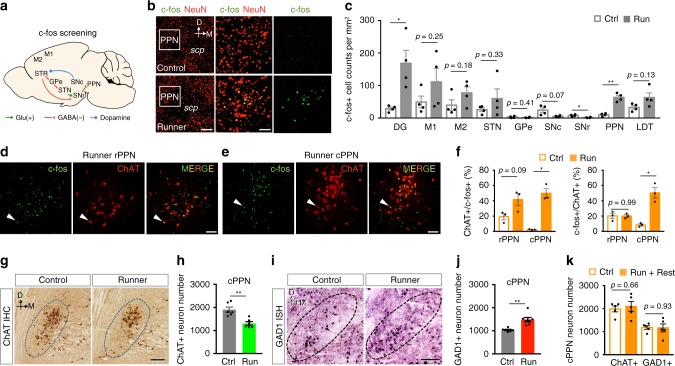
Running activates the pedunculopontine nucleus (PPN) and triggers neurotransmitter switching in the caudal PPN. **a** Motor circuitry screened for activity. **b** Double staining of neuronal marker NeuN and activity marker c-fos in a control (upper) and 1-week runner (lower). Middle and right panels are higher magnifications of the boxes in the left panels. Scp, superior cerebellar peduncle. Dorsal (D) and medial (M) axes are shown at top. Scale bar, (left) 200 μm, (middle) 50 μm. **c** c-fos immunoreactive neuron number in each region or nucleus of controls and 1-week runner mice. DG, dentate gyrus; M1, primary motor cortex; M2, secondary motor cortex; STN, subthalamic nucleus; GPe, globus pallidus external; STR, striatum; SNc, substantia nigra *pars* compacta; SNr, substantia nigra *pars* reticulata; PPN, pedunculopontine nucleus; LDT, laterodorsal tegmental nucleus. For **b**, **c**, *n* = 12 sections from 4 animals per group. **d**, **e** Double staining of ChAT and c-fos in the rostral (**d**) or caudal (**e**) PPN of a 1-week runner. White arrowheads identify examples of c-fos+ChAT+ cells. Scale bar, 100 μm. **f** Percentage of the c-fos+ChAT+ neurons in the total ChAT+ neurons (left) or in the total c-fos+ neurons (right). For **d**–**f**, *n* = 3 animals per group**. g** 3,3′-diaminobenzidine (DAB) staining of ChAT in the cPPNs of a control and 1-week runner. Dotted lines outline the PPN. Dark brown stain identifies ChAT+ neurons. Scale bar, 100 μm. **h** Stereological counts of **g**. *n* = 6 animals per group. **i** In situ hybridization of GAD1 in the cPPN of a control and 1-week runner. Scale bar, 100 μm. **j** Stereological counts of **i**. *n* = 7 animals per group. **k** Stereological counts of ChAT+ or GAD1+ neurons in the cPPN of mice that experienced 1 week of running followed by 1 week of rest and of control mice that never ran on a running wheel. *n* = 5 animals per group. Statistical significance *p < 0.05, **p < 0.01 was assessed by two-sided Welch’s *t*-test (**c**, **f**) or Mann–Whitney *U* test (other panels). Data shown are mean ± SEM.

Because transmitter switching is activity-dependent^18–20^, we first searched for c-fos expression (Fig. 2a) to determine sites of increased brain activity associated with running and to identify neurons likely to switch their transmitter. Mice that ran for 1 week exhibited a six-fold increase in the number of c-fos+ neurons in the pedunculopontine nucleus (PPN) of the midbrain compared to non-runner controls when examined immediately after the end of the last running period (Fig. 2b, c). The dentate gyrus also showed increased c-fos expression in runners, as expected^21^. The PPN was an attractive candidate for running-induced transmitter switching because it regulates gait and balance control in health^22,23^ and disease^24^. However, the rostral and caudal PPN (rPPN and cPPN) are distinct. Both contain glutamatergic, GABAergic and cholinergic neurons, but there are important differences in the proportions of their transmitter phenotypes^25^, their projection targets and activity during movement^23,26^, and their roles in behavior^27,28^. Accordingly we looked for differences in the activation of the rPPN and cPPN.

Specifically, we asked whether running activates cholinergic neurons (marked by choline acetyltransferase, ChAT, which synthesizes ACh) differently in the rPPN and cPPN, because these neurons are involved in the gait and postural disorders of Parkinson’s disease^29^. One week of running increased the number of c-fos+ neurons in both rPPN and cPPN (Supplementary Fig. 2a, b). However, running increased the percentage of c-fos+ neurons in the ChAT+ neuron population by 22-fold (from 21 of 984 to 315 of 670) in the cPPN but increased the percentage by only two-fold (from 62 of 347 to 127 of 321) in the rPPN (Fig. 2d, e, the left panel of 2f, and Supplementary Fig. 2c). Moreover, the percentage of ChAT+ neurons in the c-fos+ neuron population was not different between controls and runners in the rPPN (62 of 309 vs. 127 of 644) but increased six-fold from controls to runners in the cPPN (from 21 of 261 to 315 of 673) (Fig. 2f, right). Significantly, running increased the number of cfos+ non-cholinergic (ChAT-) neurons in the cPPN by only 1.5-fold (from 240 to 358), much less than the 15-fold (21–315) increase in the number of c-fos+ ChAT+ neurons (Supplementary Fig. 2c). These results show that cPPN cholinergic neurons are more strongly activated by sustained running than rPPN cholinergic neurons or cPPN non-cholinergic neurons, identifying cPPN cholinergic neurons as candidates for transmitter switching. The increase in the proportion of c-fos+ neurons in the ChAT+ cPPN neurons occurred after as early as 3 days of running (Supplementary Fig. 2d, e).

Indeed, chronic running for one week was accompanied by a decrease in the number of cPPN neurons expressing both ChAT (605 ± 19; Fig. 2g, h) and the vesicular acetylcholine transporter (VAChT; 459 ± 51; Supplementary Fig. 2i). This change was accompanied by an equal increase in the number of cPPN neurons expressing the gene encoding glutamic acid decarboxylase (GAD1; 551 ± 95), the enzyme that generates GABA (Fig. 2i, j). No neurogenesis or apoptosis was observed in the cPPN of either control or runner mice (Supplementary Fig. 3a–f). These results suggest that ~600 cPPN neurons switched their transmitter from ACh to GABA. There was no change in the number of ChAT+ neurons in the rPPN or the adjacent lateral dorsotegmental nucleus and no difference in the number of neurons expressing the vesicular glutamate transporter 2 (vGluT2) in the cPPN (Supplementary Fig. 3g–l).

To determine whether cholinergic cPPN activity plays a role in the running-induced ACh-to-GABA switch, we expressed Kir2.1 inward-rectifier potassium channels specifically in cPPN cholinergic neurons to suppress their activity^20^ and examined the impact on running-induced gain of GAD1 and loss of ChAT. We used a well-characterized ChAT-Cre transgenic mouse line^30,31^ that exhibited the same running-dependent loss of ChAT and gain of GAD1 expression as wild-type mice (Supplementary Fig. 2f). We found that runner mice that had received AAV-DIO-Kir2.1 showed no difference in the number of ChAT+ or GAD1+ neurons in the cPPN vs non-runner control mice and were significantly different from the runner mice that received a control AAV construct (Supplementary Fig. 2g, h). These results suggest that cholinergic cPPN activity is required for transmitter switching. Because one-third of cholinergic cPPN neurons lose ChAT, the larger fold change in c-fos expression in the cholinergic population of the cPPN compared to the rPPN (Fig. 2d, e) is caused by both an increase of c-fos and decrease of ChAT expression. Considering that two-thirds of cholinergic cPPN neurons do not switch their transmitter, the predominant increase in c-fos expression in ChAT+ neurons (Supplementary Fig. 2c) is consistent with cell-population-autonomous and non-cell-autonomous activity-dependence of transmitter switching previously reported^19^.

Mice that had run for 1 week, not subjected to motor skill training, and allowed 1 week of rest now exhibited the same number of ChAT+ and GAD1+ neurons as control mice that had never run on a running wheel (Fig. 2k). No apoptosis or neurogenesis was detected in the cPPN of these mice (Supplementary Fig. 3c–f), indicating that the transmitter switch had spontaneously reversed. The time during which the transmitter switch reversed (Fig. 2k) corresponds to the time during which the benefit of running on motor skill acquisition disappeared (Fig. 1j, k), indicating a temporal correlation between transmitter switching and the ability for enhanced motor skill learning. This finding raised the possibility that the transmitter switch is necessary for running to enhance motor skill learning. Note that the transmitter switch had reversed one week after running (Supplementary Fig. 2j), even when training and testing on the rotorod and balance beam occurred immediately after running, while the acquired motor skills persisted for at least 2 weeks (Fig. 1g–i). These results suggest that the transmitter switch is not necessary for maintaining the acquired motor skills.

The number of neurons expressing ChAT was inversely correlated with the number of neurons expressing GAD1 and directly correlated with the enhanced learning of motor skills (Supplementary Fig. 4a–e), suggesting that the level of transmitter switch determines the enhancement level of motor skill learning. The amount of running was not correlated with the motor skill acquisition (Supplementary Fig. 4f–i), perhaps because the variation in running duration is small (coefficient of variation, 0.17). Running is a categorical rather than a continuous variable and enables individual variability in transmitter switching and motor skill learning.

**Fig. 3:**
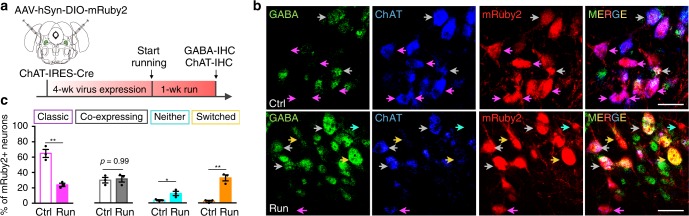
A subset of cholinergic cPPN neurons lose ChAT and gain GABA. **a** Experimental strategy to permanently label cholinergic cPPN neurons with mRuby2 fluorescent protein by expressing Cre-dependent AAV-DIO-mRuby2 in ChAT-Cre mice and determine whether the loss of ChAT and gain of GABA occur in mRuby2+ neurons. The coronal brain section was drawn according to the Franklin & Paxinos brain atlas^57^. **b** Double immunostaining of GABA and ChAT in the cPPN of a control and a 1-week runner ChAT-Cre mouse expressing AAV-DIO-mRuby2 in the cPPN. Purple arrows, mRuby2 neurons that express ChAT but not GABA (classic ChAT neurons). Gray arrows, mRuby2 neurons that express both ChAT and GABA (co-expressing neurons). Yellow arrows, mRuby2 neurons that express GABA but not ChAT (switched neurons). Cyan arrow, a mRuby2 neuron that expresses neither GABA nor ChAT. Scale bar, 50 μm. **c** The percentage of each class of mRuby2+ neurons (*n* = 844 for controls and 738 cells for runners). For **b**, **c**, *n* = 3 animals per group. Statistical significance *p < 0.05, **p < 0.01 was assessed by Mann–Whitney *U* test. Data shown are mean ± SEM.

To seek more direct evidence for neurotransmitter switching, we selectively tagged cholinergic neurons with genetic markers to reveal their change in transmitter identity following the exercise challenge. We injected a Cre-dependent AAV vector (AAV-DIO-mRuby2) into the cPPN of ChAT-Cre mice to permanently label cholinergic neurons with mRuby2, including those that subsequently lose ChAT after running (Fig. 3a, b). We then scored the number of mRuby2+ neurons that express ChAT and/or GABA immunofluorescence. The anti-GABA antibodies were validated in the superior colliculus where immunostaining of GAD67 identifies cell bodies (Supplementary Fig. 5). The colocalization between GABA and GAD67 demonstrates the specificity of the anti-GABA antibodies to detect GABAergic neurons. Co-labeling of mRuby2, ChAT and GABA revealed that in control mice, 65% (548/844) of cPPN cholinergic neurons tagged by mRuby2 expressed only ChAT, while 29% (245/844) of them co-expressed ChAT and GABA, 4% (34/844) expressed neither and 2% (17/844) expressed only GABA (Fig. 3c). In runners, 24% (177/738) of mRuby2+ neurons expressed only ChAT, 31% (229/738) co-expressed ChAT and GABA, 12% (89/738) expressed neither and 33% (243/738) expressed only GABA (Fig. 3c). The decrease in neurons expressing only ChAT and increase in neurons expressing only GABA in the ChAT-Cre line identify the expression of GABA in formerly cholinergic neurons. The increase in the number of neurons that express neither ChAT nor GABA suggests that switching neurons lose ChAT before gaining GABA. The apparent constancy of the percent of co-expressing neurons may indicate that switching and co-expressing populations are distinct.

### Transcript levels of transmitter synthetic enzymes change

**Fig. 4:**
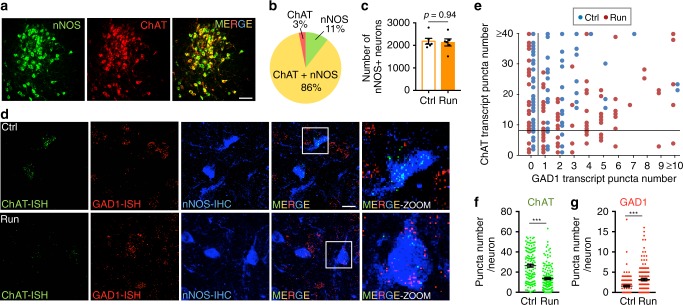
A subset of cPPN nNOS neurons of runner mice lose ChAT transcripts and gain GAD1 transcripts. **a** Double staining of the cPPN in a non-runner control mouse for nNOS (green) and ChAT (red). Scale bar, 100 μm. **b** Co-localization of nNOS and ChAT in the cPPN. For **a**, **b**, *n* = 1068 cells from three non-runner control mice. **c** Stereological counts of DAB staining of nNOS in control and 1-week runner cPPNs. *n* = 6 animals per group. Mann–Whitney *U* test. NS, not significant. **d** Triple staining of ChAT and GAD1 transcripts and nNOS protein in the cPPN of a control and a 1-week runner mouse. Right panels are boxed regions in merged images at higher magnification. Scale bar, 20 μm. **e** Scatterplot of numbers of in situ stained ChAT puncta (*y*-axis) against numbers of in situ stained GAD1 puncta (*x*-axis). Each dot represents one neuron. The vertical line divides neurons that contain zero from those containing more GAD1 transcript puncta and the horizontal line divides neurons that contain less from those containing more than eight ChAT transcript puncta. **f**, **g** Y-axes are the mean number of ChAT (**f**) and GAD1 (**g**) fluorescent puncta in single nNOS+ neurons. For **d**–**g**, *n* = 4 animals per group; *n* = 123 cells for Ctrl and 137 cells for Run. Statistical significance *p < 0.05, ***p < 0.001 was assessed by two-sided Welch’s *t*-test. Data shown are mean ± SEM.

To understand whether the loss of ChAT and gain of GAD1 occur in the same population of neurons at the transcript level, we used sensitive fluorescence in situ hybridization to analyze the levels of mRNA of transmitter synthetic enzymes in cPPN neurons from runners and controls. We used the expression of neuronal nitric oxide synthase (nNOS) as a biomarker restricted to ChAT+ neurons in the PPN^32^ (Fig. 4a, b). Although the number of ChAT+ neurons decreased with one week of running (Fig. 2g, h), the number of nNOS+ neurons did not change (Fig. 4c). We then combined immunofluorescent labeling of nNOS with RNAscope to detect mRNA encoding ChAT and GAD67 in nNOS+ neurons. By measuring the number of transcript puncta (Fig. 4d–g) and the total fluorescent area and fluorescence intensity per cell (Supplementary Fig. 6), we demonstrated a decrease in the number of ChAT transcripts and an increase in the number of GAD1 transcripts in neurons expressing nNOS in runners. While RNAscope revealed the presence of ChAT transcripts in all nNOS neurons in controls (Fig. 4e), immunostaining identified 11% of nNOS neurons that did not show detectable ChAT immunoreactivity (Fig. 4b). These results imply that in nNOS neurons of controls, the bottom 11% of the distribution of ChAT mRNA puncta (Fig. 4e; expressing <8 puncta) lack detectable ChAT immunostaining. We next grouped neurons into the four categories (as in Fig. 3) on the basis of ChAT and GAD1 mRNA expression (see Methods). In control mice, 45% of the cPPN nNOS+ cells were classic cholinergic neurons, while 44% of them co-expressed ChAT and GAD1, 6% expressed neither and 5% expressed GAD1 (Fig. 4e). However, in runners, the percentages changed to 18% for classic cholinergic neurons, 43% for co-expressing cells, 12% for neither and 27% that expressed GAD1 (Fig. 4e). These changes in the four categories are comparable to those identified by immunocytochemistry (Fig. 3c), further supporting a running-induced switch from ChAT to GAD1 in nNOS neurons. More modest co-expression of ChAT and GAD transcripts has been observed in the adult rodent PPN^33–35^; the difference is likely to result from different ways to detect and score a positive neuron using thresholding probes (see Methods). The RNAscope assay reveals that switching involves up and down regulation of transcript levels and may not entail complete disappearance and de novo appearance of transcripts of transmitter synthetic enzymes.

### Switching neurons project to the SN, VTA, and thalamus

**Fig. 5:**
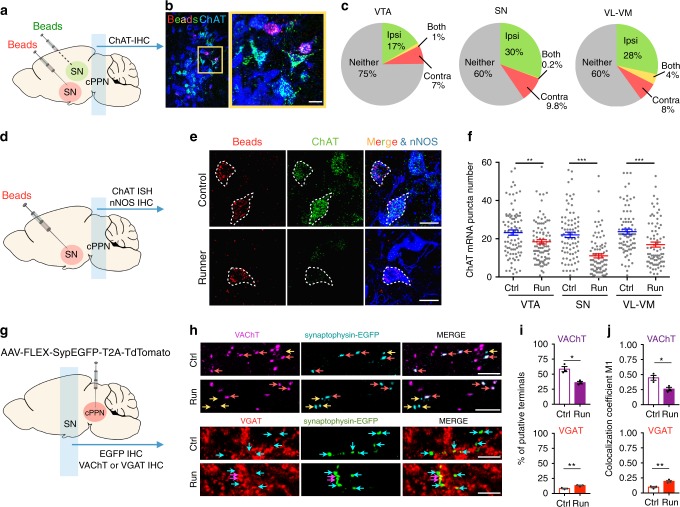
Running reduces the number of ChAT transcripts in a subset of cholinergic cPPN neurons that project to the VTA, SN and VL-VM. **a** Experimental design to validate cholinergic innervation of target nuclei by the cPPN. Red or green retrobeads (beads) were injected bilaterally into target nuclei with one color per side. Substantia nigra (SN) provides an example. **b** Triple labeling of retrobeads and ChAT in a coronal section of cPPN in a mouse injected with retrobeads. Scale bar, 50 μm. **c** Summary of the percentage of cholinergic neurons (ChAT+) that project to corresponding nuclei. Ipsi, ipsilaterally. Contra, contralaterally. Both, both ipsi- and contralaterally. Neither, no retrobeads. *n* = 3 animals examined per region. n = 829 cells for the VTA, 806 cells for the SN, and 812 for the VL-VM. **d** Experimental design to identify the target(s) of neurons with decreased numbers of ChAT transcripts. SN is shown as an example. **e** Triple-labeling of retrobeads, ChAT mRNA transcripts, and nNOS proteins in both control and runner cPPNs. Scale bar, 20 μm. **f** *Y*-axis is the mean number of ChAT fluorescent puncta in single nNOS+ cells. Each dot represents one neuron. *n* = 4 animals per group. *n* = 89 cells for VTA-Ctrl, 91 for VTA-Run, 81 for SN-Ctrl, 87 for SN-Run, 82 for VL-VM- Ctrl, 80 for VL-VM-Run. **g** Experimental design to identify presynaptic changes in transporter expression of cholinergic cPPN axons in the SN. AAV8-phSyn1-FLEX-tdTomato-T2A-Syptophysin-EGFP-WPRE (AAV-FLEX-SypEGFP-T2A-TdTomato) was injected into the cPPN of ChAT-Cre mice. Coronal sections containing the SN were immunostained for EGFP and VAChT or EGFP and VGAT. **h** Double immunostaining of VAChT and EGFP or VGAT and EGFP in the SN of non-runner controls or 1-week runners injected with AAV-FLEX-SypEGFP-T2A-TdTomato. Scale bar, 10 μm. **i** Percentage of VAChT+ or VGAT+ terminals in putatively cholinergic terminals in the SN. **j** The coefficient M1 of antibody colocalization with putative cholinergic presynaptic terminals. *n* = 10139 EGFP+ terminals for VAChT-Ctrl, 9629 for VAChT-Run, 10174 for VGAT-Ctrl and 10611 for VGAT-Run. For each group, data were quantified from 12 z-stack images from three animals. Statistical significance *p < 0.05, **p < 0.01, ***p < 0.001 was assessed by two-sided Welch’s *t*-test. Data shown are mean ± SEM.

To further test the link between neurotransmitter switching and motor skill learning, we identified targets innervated by the switching cholinergic neurons. PPN neurons project to the substantia nigra (SN), the ventral tegmental area (VTA) and the ventrolateral-ventromedial nuclei of the thalamus (VL-VM), all of which regulate motor skill learning^36–41^. Anterograde tracing of mRuby2 in ChAT-Cre neurons (see Methods) followed by retrograde tracing with retrobeads demonstrated synaptic connections between cholinergic cPPN neurons and neurons in the SN, VTA and VL-VM (Fig. 5a–c and Supplementary Fig. 7a–c). cPPN neurons originating projections to all three targets had lower mean numbers of ChAT transcript puncta in runners compared to controls, as demonstrated by triple labeling of retrograde beads, ChAT transcripts and nNOS protein (Fig. 5d–f and Supplementary Fig. 7d, e). As noted above, nNOS neurons expressing <8 puncta appear to be those lacking ChAT immunoreactivity. In controls, the percentages of neurons that express less than 8 ChAT transcript puncta and project to the SN, VTA, and VL-VM were 11%, 8 and 6%, and increased to 42%, 11 and 19% in runners. Neurons in the increased percentages of cells expressing low numbers of ChAT-transcript puncta are likely to include those that switch transmitters. The greater increase in number of the low-transcript neurons projecting to the SN (37 for Run vs. 9 for Ctrl) suggests that cPPN neurons switching transmitters make a major projection to the SN with smaller projections to the VTA (10 for Run vs. 7 for Ctrl) and VL-VM (15 for Run vs. 5 for Ctrl) (Fig. 5f).

To determine whether running up-regulates the level of vesicular GABA transporter at the cholinergic cPPN terminals in the SN, we injected an AAV vector expressing Cre-dependent synaptophysin fused with EGFP (AAV8-phSyn1(S)-FLEX-tdTomato-T2A-SypEGFP-WPRE) into the cPPN of ChAT-Cre mice to label the cholinergic cPPN terminals. Immunostaining of the cPPN with anti-tdTomato and anti-ChAT antibodies demonstrated expression of the construct in the cholinergic cPPN neurons (Supplementary Fig. 7f). Immunostaining the SN with anti-VGAT or anti-VAChT antibodies identified an increase in the number of EGFP+ putative terminals labeled with VGAT and a decrease in those labeled with VAChT in runners compared to control mice (Fig. 5g–j). These results suggest that the ACh-to-GABA switch observed in cPPN neuronal cell bodies extends to their presynaptic terminals.

### Enhanced motor skill learning requires transmitter switch

**Fig. 6:**
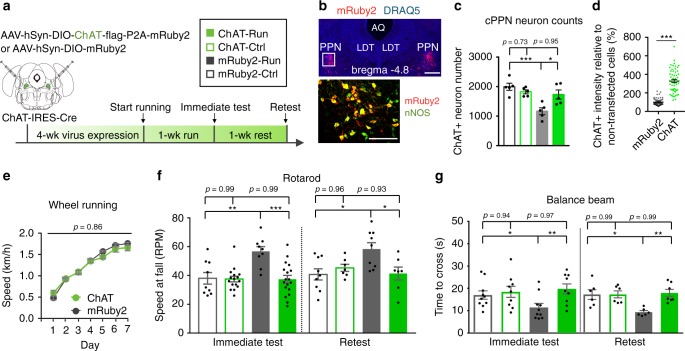
Loss of ChAT in cholinergic cPPN neurons is necessary for running-enhanced motor skill learning. **a** Experimental design to override the loss of ChAT in ChAT-Cre neurons and determine behavioral relevance. The coronal brain section was drawn according to the Franklin and Paxinos brain atlas^57^. **b** Top: mRuby2 and nuclear marker DRAQ-5 show mRuby2 expression in a coronal cPPN section of an animal bilaterally injected with AAV-DIO-ChAT-P2A-mRuby2 constructs. Coordinates adapted to Allen Brain Atlas. AQ, aqueduct. LDT, laterodorsal tegmental nucleus. Scale bar, 500 μm. Bottom: double-labeled image of nNOS and mRuby2 in boxed region of the upper image. Scale bar, 200 μm. *n* = 34 animals. **c** ChAT+ neuron number in the cPPN of runners and non-running controls that were injected with AAV-DIO- mRuby2 or AAV-DIO-ChAT. *n* = 5 animals per group. Nonparametric Kruskal–Wallis test followed by Dunn’s correction. **d** ChAT fluorescence intensity for mRuby2-expressing cells in ChAT-Cre mice injected with AAV-DIO-ChAT or AAV-DIO-mRuby2. Fluorescence intensity was normalized by non-transfected cells (mRuby2 negative). *n* = 56 and 58 cells for mRuby2 and ChAT. *n* = 3 animals per group. Welch’s *t*-test. **e** Wheel running speed of mice that were injected with AAV-DIO-ChAT or AAV-DIO-mRuby2 at the cPPN. *n* = 10 animals per group. Linear regression followed by two-sided Welch’s *t*-test. **f**, **g** Rotarod (**f**) and balance beam (**g**, 4 mm rod) tests show that expression of mRuby2 did not affect enhancement of acquisition and maintenance of motor skills gained by running (mRuby2-Run vs. mRuby2-Ctrl), whereas exogenous ChAT expression blocked the enhancement (ChAT-Run vs. mRuby2-Run). The blockade was sustained after rest for 1 week. For immediate rotarod test, numbers of animals are 9 for mRuby2-Ctrl, 16 for ChAT-Ctrl, 9 for mRuby2-Run, 18 for ChAT-Run. For rotarod retest, numbers of animals are 9 for mRuby2-Ctrl, 6 for ChAT-Ctrl, 9 for mRuby2-Run, 7 for ChAT-Run. For immediate test of balance beam, numbers of animals are 10 for mRuby2-Ctrl, 9 for ChAT-Ctrl, 10 for mRuby2-Run, 9 for ChAT-Run. For balance beam retest, numbers of animals are 6 for mRuby2-Ctrl, ChAT-Ctrl, mRuby2-Run and 7 for ChAT-Run. ANOVA followed by Tukey’s test. *p < 0.05, **p < 0.01, ***p < 0.001. Data shown are mean ± SEM.

**Fig. 7:**
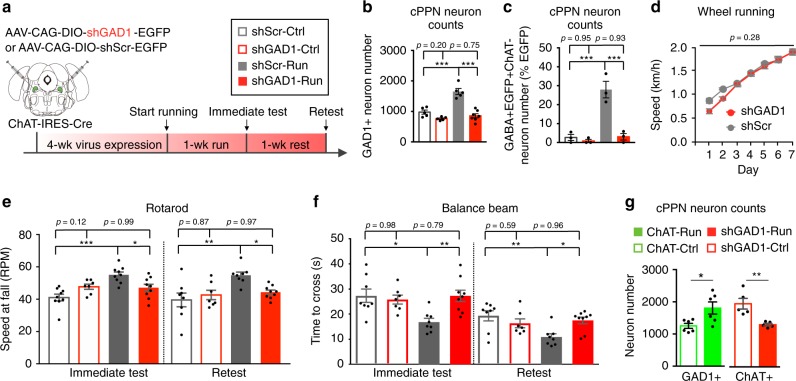
Gain of GAD1 in cholinergic cPPN neurons is also necessary for running-enhanced motor skill learning. **a** Experimental design to override the gain of GAD1 in ChAT-Cre neurons and examine behavioral relevance. The coronal brain section was drawn according to the Franklin and Paxinos brain atlas^57^. **b** Numbers of GAD1 in situ stained neurons in the cPPNs of runners and non-running controls that were injected with AAV-DIO-shScr (scramble shRNA) or AAV-DIO-shGAD1 (shRNA for GAD1). *n* = 7 for shGAD1- Run, and five animals for the other three groups. Nonparametric Kruskal–Wallis test followed by Dunn’s correction. **c** Percentage of neurons immunostained as GABA+, EGFP+ and ChAT- in the total EGFP+ neurons in cPPNs of ChAT-Cre runners and non-running controls that were injected with AAV-DIO-shScr or AAV-DIO-shGAD1. *n* = 1243 cells for shScr-Ctrl, 1258 for shScr-Run, 957 for shGAD1-Ctrl, and 1226 for shGAD1-Run from three animals per group. Nonparametric Kruskal–Wallis test followed by Dunn’s correction. **d** Wheel running speed of ChAT-Cre mice that express AAV-DIO-shScr or AAV-DIO-shGAD1 in the cPPN. *n* = 8 animals per group. Two-sided Welch’s *t*-test. **e**, **f** Rotarod (**e**) and balance beam (**f**, 4-mm rod) tests show that expression of shScr did not affect the enhancement of acquisition and maintenance of motor skills gained by running (shScr-Ctrl vs. shScr-Run) whereas knocking down GAD1 blocked the enhancement (shGAD1-Run vs. shScr-Run). Blockade was sustained after rest for 1 week. For **e**, animal numbers are 9 for shScr-Ctrl, 7 for shGAD1-Ctrl, 9 for shScr-Run and 9 for shGAD1-Run for immediate test and 8 for shScr-Ctrl, 7 for shGAD1-Ctrl, 8 for shScr-Run and 9 for shGAD1-Run for retest. For **f**, numbers of animals are 8 for shScr-Ctrl, 7 for shGAD1-Ctrl, 8 for shScr-Run, and 9 for shGAD1-Run for both immediate test and retest. **g** Numbers of GAD1 in situ stained neurons in the cPPN of 1-week runners and non-runner controls that express AAV-DIO-ChAT and numbers of ChAT immunostained neurons in 1-week runners and non-runner controls that express AAV-DIO-shGAD1. *n* = 6 animals per group for ChAT-Run and ChAT-Ctrl and 5 for shGAD1-Run and shGAD1-Ctrl. Mann–Whitney *U* test. *p < 0.05, **p < 0.01, ***p < 0.001. Data shown are mean ± SEM.

To determine whether neurotransmitter switching is necessary for the beneficial effect of running on motor skill learning, we injected AAV-DIO-ChAT into the cPPN of ChAT-Cre mice to continuously express ChAT in all cholinergic neurons (Fig. 6a, b and Supplementary Fig. 8a–d). The ChAT-Cre line^30,31^ demonstrated the same running-dependent transmitter switch as wild-type mice, even with expression of control constructs (AAV-DIO-mRuby2, Fig. 6c; AAV-DIO-shScr, Fig. 7b). Overexpression of ChAT did not change the number of ChAT+ neurons in the cPPN of control ChAT-Cre mice and maintained the number of ChAT+ neurons at control levels after sustained running (Fig. 6c). Although the overexpression of ChAT caused a 3-fold increase in the level of ChAT expression in control mice (Fig. 6d and Supplementary Fig. 8c), it did not affect their basal motor skill learning (Fig. 6f, g and Supplementary Fig. 9e, f) or running activity in runner mice (Fig. 6e and Supplementary Fig. 9a, b). This may result from feedback inhibition of ChAT by ACh^42,43^ that is likely to maintain ACh levels of the non-switching neurons in the physiological range. Mice that had received AAV-DIO-ChAT acquired the same running skill as wild-type mice or ChAT-Cre mice injected with AAV-DIO-mRuby2 but their motor learning on the rotarod and balance beam, tested directly after 1 week of running, was not enhanced (Fig. 6f, g). The slopes of the learning curves for both rotarod and balance beam behaviors were significantly steeper for runner mice expressing AAV-DIO-mRuby2 (10 ± 2 rpm/trial and −3.9 ± 1.0 s/trial) than for runners expressing the AAV-DIO-ChAT (6 ± 1 rpm/trial, −1.3 ± 0.4 s/trial; *p* = 0.037 and *p* = 0.035) and test performances of runners expressing AAV-DIO-mRuby2 were significantly better (Supplementary Fig. 9e, f). Overriding the loss of ChAT also prevented enhancement of motor skill learning when mice ran, were trained and tested, rested for one week and then re-tested (Fig. 6f, g). This finding makes it unlikely that exogenous expression of ChAT had simply delayed the improvement in motor skill learning.

Expression of shRNA for GAD1 (AAV-DIO-shGAD1) to suppress the gain of GAD67 in cholinergic cPPN neurons similarly prevented improved motor learning following one week of running, compared to mice expressing a Cre-dependent scrambled shRNA sequence (AAV-DIO-shScr; Fig. 7a–f, Supplementary Figs. 8 and 9). Knockdown of GAD1 maintained the number of GAD1+ neurons at control levels in the cPPN after sustained running (Fig. 7b). Because immunostaining of GAD67 in the PPN detects a large number of synaptic puncta that makes it challenging to analyze cell bodies, the knockdown efficiency of GAD67 expression by shGAD1 was tested in the motor cortex and measured to be 65% (Supplementary Fig. 8e, f). Moreover, shGAD1 blocked the increase in the expression of GABA in cholinergic cPPN neurons of runner mice (Fig. 7c), indicating that GAD1 has the dominant role in regulating the level of GABA in these neurons and that shGAD1 effectively prevents the gain in GABA caused by running. Suppressing the gain of GAD1 did not affect acquisition of the wheel running skill (Fig. 7d and Supplementary Fig. 9c, d) but motor learning on the rotarod and balance beam, tested directly after one week of running, was not enhanced for a period that was extended to one week of rest (Fig. 7e,f). The slopes of the learning curves were again steeper for runner mice expressing AAV-DIO-shScr (9 ± 1 rpm/trial, −6.7 ± 0.9 s/trial) than for runners expressing AAV-DIO-shGAD1 (5 ± 2 rpm/trial, −3.7 ± 1.0 s/trial; *p* = 0.073 and *p* = 0.041) and test performances for runners expressing AAV-DIO-shScr were again significantly better (Supplementary Fig. 9g, h). These results suggest that both the loss of ACh and gain of GABA are required for enhancement of motor skill learning. Overriding the loss of ChAT or suppressing the gain of GAD1 did not affect running-induced c-fos expression in cholinergic cPPN neurons (Supplementary Fig. 10). We tested for population-level control of the change in expression of one transmitter by overriding the change in expression of the other. Notably, suppressing the gain in GAD1 expression did not affect the loss of ChAT expression and vice versa (Fig. 7g). Although expression of these two transmitter synthetic enzymes is inversely correlated, the two are not reciprocally regulated.

## Discussion

**Fig. 8:**
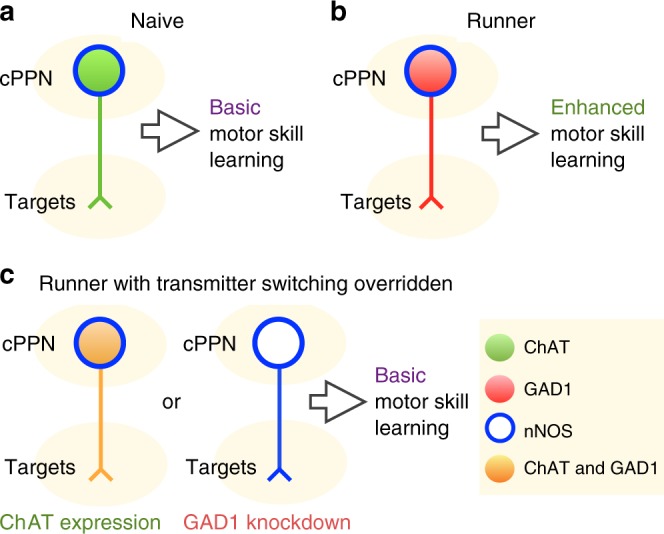
Transmitter switching in the cPPN regulates motor skill learning. **a**, **b** Chronic running induces neurotransmitter switching from ACh to GABA in the cPPN and enhances motor skill learning. **c** Both ChAT loss and GAD1 gain are necessary for running-enhanced motor skill learning.

Our findings provide insight into the mechanism by which sustained running improves acquisition of motor skills (Fig. 8). The functional significance of ACh-to-GABA transmitter switching is demonstrated by changes in behavior that are reversed by overriding the switch. Switching involves changes in levels of transcripts of transmitter synthetic enzymes. Activity-dependent transcription factor phosphorylation^18,19^ and microRNA regulation^44^, which have been implicated in transmitter switching in the developing nervous system, are candidates for implementing the switch in the adult CNS. Transmitter switching in the cPPN, particularly when it may change the sign of the synapse from excitatory to inhibitory, appears to rewire motor circuitry to enhance motor skills. Acquisition of motor skills requires many components including balance, coordination, motivation, attention and muscle strength. Interestingly, the PPN regulates many of them^23,45^, and transmitter switching in the cPPN may contribute to motor skill learning through regulating one, several or even all of these aspects. The persistence of learned behaviors after the transmitter switch has reversed implies that there is continued capacity for plasticity in locomotor circuitry.

We examined three targets of the cPPN and found that cholinergic cPPN neurons projecting to each of the three targets showed a significant reduction of ChAT transcripts (Fig. 5). This suggests that the reduction of ChAT expression might be observed in cholinergic cPPN neurons projecting to other targets as well. Multi-target regulation is not surprising because cholinergic PPN neurons have an average of five axonal collaterals that allow a single cholinergic neuron to project to many targets^23^. Cholinergic cPPN neurons may regulate motor function and other behaviors by gating the activity of downstream targets through transmitter switching.

Dopaminergic and noradrenergic neurons are implicated in fine motor function plasticity that regulates paw and digit movements^46–49^. Our results unexpectedly indicate that acquisition of high-demand rotarod and balance beam performance requires transmitter switching in cholinergic cPPN neurons. Overriding transmitter switching did not affect the ability to run on a running wheel (Figs. 6 and 7), perhaps because running is not an exceptionally taxing motor skill. Consistent with these results, selective lesions of cholinergic PPN neurons impair learning of high-demand running on an accelerating-speed rotarod but do not affect learning of low-demand running on a fixed-speed rotarod or basal locomotion^50^. In contrast, glutamatergic and GABAergic PPN neurons regulate gait and speed of locomotion^51–53^.

Control of voluntary movement and procedural learning are core functions of the basal ganglia. SNr inhibitory neurons serve as the major output of the basal ganglia and project to the PPN; in turn, PPN ascending axons project to the SNc and SNr to modulate the function of the basal ganglia^23^. We propose a model in which conversion of cPPN excitatory cholinergic input to inhibitory GABAergic input of inhibitory neurons in the SN enables feedback control of SN neurons regulating motor coordination and skill learning, as supported by their decrease in c-fos expression (Fig. 2c). Gait speed, stride and balance of Parkinson’s disease and stroke patients are improved following sustained treadmill training^54–56^. Our finding that transmitter switching after running is a critical event for improving motor skill learning suggests that transmitter switching may be important in many circumstances where sustained exercise benefits behavior.

## Methods

### Mice

All animal procedures were carried out in accordance with NIH guidelines and approved by the University of California, San Diego Institutional Animal Care and Use Committee or Scripps Institutional Animal Care and Use Committee. C57BL/6J (JAX#000664) mice were obtained from Jackson Laboratories. ChAT-IRES-Cre (JAX#006410) mice were obtained from the Byungkook Lim lab and Jackson Laboratories. PV-IRES-Cre (JAX#008069) mice were provided by the Stefan Leutgeb lab. Animals were maintained on a 12 h:12 h light:dark cycle (light on: 10:00 pm–10:00 am) with food and water ad libitum. Vivarium temperature was between 65 and 75 °F (~18 and 23 °C) with 40–60% humidity. The ChAT-IRES-Cre colony was maintained by breeding homozygous male ChAT-Cre mice with female wild-type C57BL/6J mice. Heterozygous ChAT-Cre offspring were used in the study. Both heterozygous and homozygous PV-IRES-Cre mice were used. All experiments were performed on 8- to 12-week-old male mice.

### Wheel running

Mice were single-housed in hamster cages and provided with FastTrac (Bio-serv, K3250) or digital (Med Associates ENV-044) running wheels that are identical in shape and size. Mice were allowed voluntary running for one week and were continuously recorded with Swann DVR4-2600 infrared video cameras. Control mice were housed with running wheelbases without wheels. Episode duration and time with the wheel were hand-scored using JWatcher software. The maximum angular excursion of mouse movements on the running wheels was plotted using Image J and measured with a protractor. The digital running wheels recorded running distance and running speed was calculated as distance divided by time.

### Rotarod

Training and tests were performed as the rotarod (Ugo Basile, 57624) accelerated from 5 rpm to 80 rpm in 6 min. The rpm at which mice fell off was recorded by the rotarod. Mice were trained for nine trials on the first day, followed by three tests the next day and three retests after 1, 2, or 4-weeks rest. There was a 10-min interval between each trial/test. The single digit accuracy of the slopes of learning curves reflects the accuracy of recording the rpm at fall by the rotarod.

### Balance beam

Mice were trained and tested with balance beams 1 meter long and 0.75 meters above the floor. Twelve millimeters and 6 mm square beams (S-12 and S-6) were single plastic horizontal bars while 6 and 4 mm rod beams (R-6 and R-4) consisted of two stainless steel parallel bars 5 cm apart. On the training day mice were trained to cross S-12 for three trials, S-6 for three trials, R-6 for three trials and then R-4 for three trials, each 10 min apart. On the test and retest day mice were allowed to cross all four beams in the same order, three times for each, 10 min apart. A video camera was installed at the end of the beam where mice started walking. At the other end of the beam, a black box with an entry facing the beam was installed to attract mice to cross the beams. Time to cross the beam and enter the escape box was hand-scored and the investigator was double-blinded to the history of the mice. The slopes of learning curves are accurate to one decimal place because the time to cross the beam was hand scored and human response time is 0.1~0.2 s.

### Motor skill learning

The mean speed at fall from a rotarod for the nine trials on the training day was fitted by a one-phase association model. Mean data points for each training trial were plotted and fitted using GraphPad Prism 7 software and the coefficient of determination (*R*^2^) was used to justify the fit (*R*^2^ > 0.96 for controls and *R*^2^ > 0.95 for runners, with MATLAB). The mean time to cross a 4 mm rod beam for the three trials on the training day was fitted by linear regression (*R*^2^ > 0.98 for controls and *R*^2^ > 0.99 for runners, with MATLAB).

### Locomotor activity

Mice were tested for 120 min in polycarbonate cages (42 × 22 × 20 cm) placed in frames (25.5 × 47 cm) mounted with two levels of photocell beams at 2 and 7 cm above the bottom of the cage (San Diego Instruments, San Diego, CA). The two sets of beams detect both horizontal (roaming) and vertical (rearing) behavior. A thin layer of bedding material covered the bottom of the cage. Data were collected in 1-min epochs.

### Histology and immunocytochemistry

Animals were perfused transcardially with phosphate-buffered saline (PBS) followed by 4% paraformaldehyde (PFA) in PBS right after the last episode of running, i.e., mice started to run at 10 am at light-off and kept running until they were perfused between 2 and 3 pm. Brains were dissected and post-fixed in 4% PFA for 16 to 24 h at 4 °C, washed in PBS for 1 min and transferred to 30% sucrose in PBS for 2 days at 4 °C. Forty-micrometers coronal sections were cut on a microtome (Leica SM2010R) and stained.

For immunostaining, sections were permeabilized and blocked in 24-well culture plates for 2 h in a blocking solution (5% normal horse serum, 0.3% Triton X-100 in PBS) at 22–24 °C. Primary and secondary antibodies were diluted in the blocking solution. Incubation with primary antibodies was performed for 48 h on a rotator at 4 °C. After washing in PBS (three times, 15 min each), secondary antibodies were added for 2 h at 22–24 °C. For immunofluorescence, sections were mounted with Fluoromount-G (Southern Biotech) or ProLong Gold Antifade Mountant (Life Technologies) containing DRAQ-5 (Thermo Fisher, 62251, 1:1000 dilution; when nuclear staining was needed) after washes in PBS (three times, 15 min each).

For DAB (3,3′-Diaminobenzidine) staining, sections were treated with 0.3% hydrogen peroxide for 30 min, washed in PBS (three times, 5 min each), incubated with Vectastain Elite ABC HRP mixture (Vector Laboratories, PK-6100) for 45 min, washed in PBS (three times, 15 min each), and signals were developed using the DAB Peroxidase Substrate kit (Vector Laboratories, SK-4100).

Primary antibodies used in this study were goat anti-ChAT (Millipore, AB144P, 1:500), rabbit-anti-nNOS (Thermo Fisher, 61-7000, 1:500), goat-anti-cFos (Santa Cruz, sc-52G, 1:300), rabbit-anti-cFos (Santa Cruz, sc-52, 1:300), mouse-anti-cFos (Abcam, ab208942, 1:500), rabbit-anti-PV (Swant, PV25, 1:2000), goat-anti-VAChT (Millipore, ABN100, 1:500), goat-anti-VGAT (Synaptic system, 131004, 1:1000), mouse-anti-NeuN (Millipore, MAB377, 1:500), rabbit-anti-GABA (Sigma-Aldrich, A2052, 1:1000) rabbit-anti-GFP (Thermo Fisher, A11122, 1:1000), chicken anti-GFP (Abcam, ab13970, 1:1000), guinea pig anti-GFP (Synaptic Systems, 132005, 1:3000), rabbit-anti-zsGreen (Takara, 632474, 1:500), goat-anti-doublecortin (Santa Cruz, sc-8066, 1:300) and rabbit-anti-Ki67 (Cell Signaling, 9129, 1:300). Secondary antibodies for immunofluorescence were from Jackson ImmunoResearch Labs and used at a concentration of 1:600: Alexa Fluor-488 donkey-anti-rabbit (705-545-003), Alexa Fluor-488 donkey-anti-guinea pig (706-545-148), Alexa Fluor-488 donkey-anti-mouse (715-545-150), Alexa Fluor-488 donkey-anti-goat (705-545-147), Alexa Fluor-594 donkey-anti-goat (705-585-147), Alexa Fluor-594 donkey-anti-mouse (715-585-150), Alexa Fluor-647 donkey-anti-goat (705-605-147) and Alexa Fluor-647 donkey-anti-rabbit (711-605-152). Biotinylated goat anti-rabbit (BA-1000) and horse anti-goat (BA-9500) secondary antibodies for DAB staining were from Vector Laboratories and used at a concentration of 1:300.

In situ hybridization PFA fixed brains were dissected, post-fixed in 4% PFA for 16 to 24 h at 4 °C and transferred to 30% DEPC-sucrose in PBS for 2 days. Subsequently, brains were embedded in 30% DEPC-sucrose and frozen with dry ice. Forty-micrometers cryosections were collected on Superfrost Plus slides (VWR, 48311-703) and used for mRNA in situ hybridization. Complementary DNAs (cDNAs) of *gad1* or *slc17a6* (sequences from Allen Brain Atlas: *gad1*, RP_040324_01_F01; *slc17a6*, RP_050921_01_E03) were cloned from mouse cDNA library in ~800-base-pair segments into a pGEM vector and sequenced to verify that they were correct. Antisense complementary RNA (cRNA) probes were synthesized with T7 (Promega, P2075) or Sp6 polymerases (Promega, P1085) and labeled with digoxigenin (Roche, 11175025910). Hybridization was performed with 1 to 5 μg/ml cRNA probes at 65 °C for 20 to 24 h. Probes were detected using Anti-Digoxigenin-AP Fab fragments (Roche, 11093274910, 1:5000). Signals were developed for 8 h using a mixture of 4-Nitro blue tetrazolium chloride (Roche, 11383213001) and BCIP 4-toluidine salt solution (Roche, 11383221001). We counted all GAD1+ neurons in the cPPN, including those that looked intense, moderate or faint.

Fluorescent RNAscope in situ hybridization was performed according to the manufacturer’s instructions (Advanced Cell Diagnostics) with some modifications: In an RNase-free environment, 12-μm fixed brain sections were mounted on Superfrost Plus slides immediately after microtome sectioning and air-dried in a 60 °C oven for 30 min. Sections were rehydrated in PBS for 2 min and incubated for 5 min in 1× target retrieval solution at 95 °C. Sections were then rinsed with distilled water for 5 s and rinsed in 100% ethanol for 5 seconds. After air-drying, sections were incubated with the following solutions in a HybEZ humidified oven at 40 °C with three rinsing steps in between each: protease III, 30 min; probes, 2 h; amplification (Amp) 1-fluorescence (FL), 30 min; Amp 2-FL, 15 min; Amp 3-FL, 30 min; and Amp 4-FL, 15 min. Ready-to use Amp 1-FL, Amp 2-FL, Amp 3-FL, Amp 4-FL and 50× washing solution for rinsing steps were included in the RNAscope Multiplex Fluorescent Reagent kit. Standard immunofluorescent staining was subsequently performed in the dark^15^. Probes for mouse *chat* mRNA (Catalog number: 408731-C2) and *gad1* mRNA (Catalog number: 400951) were from Advanced Cell Diagnostics. Sections were 6–7 μm thick post-processing. Six optical sections (1 µm Z step) of each physical section were examined and regions of interest (ROIs) were drawn around the boundaries of nNOS cells on the optical section that showed the best focal plane for each cell (largest cross-sectional area). These ROIs were scored for ChAT and GAD1 transcript puncta using Image J. The average area of ROIs was consistent between the control and runner groups. We included negative controls using a probe targeting a bacterial-origin gene *dapB* (Catalog number: 310043; GeneBank: EF191515) in each batch of experiments and validated that the negative control group showed <1 punctum in 100 cells.

Based on the expression of ChAT and GAD1 transcript puncta, the neurons were grouped into four categories (1) classic cholinergic neurons: ≥8 ChAT transcript puncta, no GAD1 transcript puncta; (2) co-expressing neurons: ≥8 ChAT puncta, ≥1 GAD1 puncta); (3) neurons expressing neither: <8 ChAT puncta, no GAD1 puncta; (4) switched neurons: <8 ChAT puncta, ≥1 GAD1 puncta. We are aware that other studies have arrived at a lower percent of costaining than reported here. The difference is likely to be caused by different ways to detect and score a positive neuron. Detection by IHC and ISH can be greatly affected and is ultimately determined by the sensitivity and thresholding of the method. From the distribution of GAD1 puncta number (Fig. 4e) we infer that scoring only neurons that contain 5 or more GAD1 puncta, would yield an incidence of 4% of ChAT neurons that co-express GAD1, which would be consistent with percentages previously reported^33–35^. We have not found descriptions of counting thresholds in other reports and believe that in most cases investigators count neurons that contain moderate to intense GAD1 and have regarded those with a low level of transcripts as negative.

### Birthdating

Mice were intraperitoneally injected with BrdU (50 mg/kg) once every 12 h for 1 week. PFA-fixed brains were dissected, post-fixed and dehydrated as described above. Forty-micrometers cryosections were collected and treated with 1 M HCl for 30 min at 45 °C for DNA denaturation. After rinsing in PBS (three times, 5 min each), sections were incubated in a mixture of 0.3% Triton X-100 and 5% horse serum in PBS for 1 h, incubated in rat-anti BrdU antibody (AbD Serotec, MCA2060, 1:300) at 4 °C overnight, rinsed in PBS (three times, 5 min each) and amplified by Alexa Fluor-488 donkey anti-rat antibody (Life Technologies, A21208, 1:600). Sections were mounted with Fluoromount containing DRAQ-5 (1:1000).

### TUNEL assay

The In Situ Cell Death Detection (TUNEL) Kit with TMR Red (Roche, 12156792910) was used to detect in situ apoptosis. Forty-micrometers cryosections were re-fixed with 1% PFA for 20 min at 22–24 °C and rinsed with PBS (three times, 5 min each). Sections were then permeabilized in 0.1% sodium citrate and 1% Triton X-100 for 1 h at 22–24 °C. After rinsing in PBS (three times, 5 min each), sections were incubated with TUNEL reaction solution according to the vendor’s instruction, i.e., incubated in a mixture of 25 μL of terminal-deoxynucleotidyl transferase solution and 225 μL of label solution. Incubation was performed in a humidified chamber for 3 h at 37 °C in the dark. Sections were rinsed and mounted with Fluoromount containing DRAQ-5 (1:1000). For a positive control, sections were treated with DNase I (10 U/mL, New England Biolabs, M0303S) for 1 h at 37 °C and rinsed in PBS (three times, 5 min each), followed by incubation with the TUNEL mixture.

### Imaging and data analysis

Fluorescent images were acquired with a Leica SP5 confocal microscope with a 25×/0.95 water-immersion objective and a z resolution of 1 μm. Leica Application Suite X software was used for fluorescent cell counting and all sections within the confocal stacks were examined without maximal projection. RNAscope in situ signals were quantified using Image J. Exemplar images are maximum intensity projections of 6 consecutive confocal sections when RNAscope signals are included (Figs. 4d and 5e) and 16–24 confocal sections for other immunofluorescence images. For the synaptophysin-EGFP signals, images were acquired with a 63×/0.90 water-immersion objective at zoom 2 magnification with a z resolution of 1 μm. Colocalization was quantified using Image J with the JACoP plugin. Exemplar images for DAB staining and in situ hybridization were acquired with a NanoZoomer Slide scanner (Hamamatsu S360) with a 20×/0.75 air objective. Counting DAB-stained c-fos neurons per unit area was performed with Image J on images harvested from the NanoZoomer. To delimit the PPN in sections stained for c-fos (Supplementary Fig. 2a, b), or for TUNEL and Ki67/DCX (apoptosis and neurogenesis, Supplementary Fig. 3a–f), an adjacent section was stained for ChAT to define the boundaries of the PPN.

Images were analyzed exhaustively or by sampling. Exhaustive analysis entailed scoring all neurons in every other section through the entire brain region of interest and reporting the number of cells for the number of mice examined. Sampling involved scoring and reporting a specific number of cells from a specified number of sections from a particular number of mice. In these cases, exact cell and/or section numbers are shown in the figure legends.

### Stereological counting

Stereo Investigator software (MBF Bioscience) was used to exhaustively count DAB immunostained or in situ hybridization-stained cells. Counting was carried out using optical fractionator sampling on a Zeiss Axioskop 2 microscope (40×/0.65 Ph2 objective) equipped with a motorized stage. The population of midbrain PPN neurons was outlined on the basis of ChAT immunolabeling, with reference to a coronal atlas of the mouse brain^57^ and anatomical landmarks such as fiber tracts. Caudal PPN refers to the caudal half of PPN with reference to a transverse line normal to and halfway along the rostrocaudal axis; rostral PPN refers to the rostral half. To count GABAergic and glutamatergic neurons in the sections stained by in situ hybridization, an adjacent section was stained for ChAT to define the ROI for the boundaries of PPN. The area of the ROI and the cell density were compared between the control and experimental groups to ensure that the counted areas were comparable between the two groups and that changes in cell density were consistent with changes in the absolute counts. Pilot experiments with continuous counting determined that counting every other section was sufficient to estimate the number of cholinergic, GABAergic, glutamatergic and nitroxidergic neurons in the cPPN of each brain. Consequently, five to six sections were counted for each mouse brain. The average section thickness was measured prior to counting. Sections shrank from 40 μm to 22–24 μm after staining and dehydration. Exhaustive counting was performed and no sampling grid was skipped because the distribution of neurons in the cPPN is uneven. Counting was performed by two investigators double-blinded to the origin of the sections.

### Viral constructs and injection

Seven to eight-week-old male mice were anesthetized with a mixture of 120 mg/kg ketamine and 16 mg/kg xylazine and head-fixed on a stereotaxic apparatus (David Kopf Instruments Model 1900) for all stereotaxic surgeries. The caudal PPN was targeted bilaterally (cPPN: anterior–posterior (AP), −4.80 mm from bregma; mediolateral (ML), ±1.25 mm; dorsal–ventral (DV), −3.25 mm from the dura). A total of 300 nL of the Cre-on viral vectors were injected into each cPPN of ChAT-Cre mice at a rate of 100 nL/min using a syringe pump (PHD Ultra™, Harvard apparatus, no. 70-3007) installed with a microliter syringe (Hampton, no.1482452A) and capillary glass pipettes with filament (Warner Instruments, no. G150TF-4). Pipettes were left in place for 5 min after injection. Titers of recombinant AAV vectors ranged from 7.1 × 10^12^ to 2.2 × 10^13^ viral particles/ml, based on quantitative PCR analysis. AAV8-phSyn1(S)-FLEX-tdTomato-T2A-SypEGFP-WPRE was purchased from the Salk Viral Vector Core and used to trace the axonal terminals^58^. AAV-DJ-CMV-DIO-Kir2.1 and AAV-DJ-CMV-DIO-EGFP were both ordered from the Stanford Viral Vector Core and used to suppress neuronal activity or as a control^20,59^. pAAV-hSyn-DIO-ChAT-P2A-mRuby2 and pAAV-hSyn-DIO-mRuby2 plasmids were constructed in our lab and AAV2/8 vectors were packaged in the Salk Viral Vector Core. Vector Biolabs produced AAV8-CAG-DIO-shRNAmir-mGAD1-EGFP and AAV8-CAG-DIO-shRNAmir-Scramble-EGFP vectors. The shRNA sequence for mouse GAD1 is

*5*′*-GTCTACAGTCAACCAGGATCTGGTTTTGGCCACTGACTGACCAGATCCTTTGACTGTAGA-3*′.

### Tract tracing

For anterograde tracing, 300 nL of Cre-on AAV8-DIO-mRuby2 vectors were unilaterally injected into the cPPN of ChAT-Cre mice as described above and axonal tracts were imaged by Leica confocal microscope. For retrograde tracing, a volume of 100 nL of retrobeads (Red Retrobeads™ IX or Green Retrobeads™ IX, Lumafluor) was injected at 100 nL/min into the SN or VTA and VL-VM. Pipettes were left in place for 10 min after injection. Three weeks after injection, mice were sacrificed for brain examination or housed with running wheels to examine running-induced loss of ChAT transcripts using RNAscope combined with nNOS immunostaining. ChAT transcripts in neurons that contained retrobeads were quantified using Image J. Retrobead infusion coordinates were SN (from bregma AP −3.20 mm; ML ±1.50 mm; and DV from the dura, −4.00 mm), VTA (AP, −3.10 mm; ML, ±0.50 mm; and DV, −4.25 mm), and VL-VM (AP, −2.20 mm; ML, ±1.75 mm; and DV, −3.25 mm).

### Statistics and reproducibility

For comparisons between two groups, data were analyzed by Welch’s *t*-test for normally distributed data and the Mann–Whitney *U* test for data not normally distributed, using Graph Pad Prism 7. For repeated samples, data were analyzed by paired *t*-test. Correlation was analyzed by the Pearson correlation using Graph Pad Prism 7. All tests are two-tailed. Statistical analyzes of the data were performed using Prism 7 software for the number of animals for each experiment indicated in the figure legends. Means and SEMs are reported for all experiments. For comparisons between multiple groups, ANOVA followed by Tukey’s test was used for normally distributed data. The nonparametric Kruskal–Wallis test followed by Dunn’s correction was used for data that were not normally distributed. Experiments were carried out independently twice (Figs. 2b, c, 4a, b, 5b, c, h–j, 7b and Supplementary Figs. 1f, 3a–f, 5, 7f, 8f), three times (Fig. 1b, h–j, 2d–k, 3, 4c–h, 5e–f, 6c–d, 7c, g, and Supplementary Figs. 1a–d, 2b–f, i–j, 3g–l, 4a–c, f–i, 6, 7d–e, 8, 9a–d and 10), or four or more times (Figs. 1c–f, 6e–g, 7d–f and Supplementary Figs. 2g, i, 4d, e, 9e–h).

### Reporting summary

Further information on research design is available in the Nature Research Reporting Summary linked to this article.

## Supplementary information

Supplementary Information

Reporting Summary

## Data Availability

All data generated or analyzed during this study are included in this published article and its supplementary information files. Source data are provided as a Source data file.

## Source data

Source Data

## References

[1] Bostan AC, Strick PL (2018). The basal ganglia and the cerebellum: nodes in an integrated network. Nat. Rev. Neurosci..

[2] Esposito MS (2014). Brainstem nucleus MdV mediates skilled forelimb motor tasks. Nature.

[3] Dayan E, Cohen LG (2011). Neuroplasticity subserving motor skill learning. Neuron.

[4] McKenzie IA (2014). Motor skill learning requires active central myelination. Science.

[5] Statton MA (2015). A single bout of moderate aerobic exercise improves motor skill acquisition. PLoS ONE.

[6] Palmer SS (1986). Exercise therapy for Parkinson’s disease. Arch. Phys. Med. Rehabil..

[7] Kane K, Bell AA (2009). core stability group program for children with developmental coordination disorder: 3 clinical case reports. Pediatr. Phys. Ther..

[8] Rafie F (2015). Physical exercises and motor skills in autistic children. Iran. J. Public Health.

[9] Meijer JH, Robbers Y (2014). Wheel running in the wild. Proc. Roy. Soc. B.

[10] Murphy TH, Corbett D (2009). Plasticity during stroke recovery: from synapse to behaviour. Nat. Rev. Neurosci..

[11] Petzinger GM (2013). Exercise-enhanced neuroplasticity targeting motor and cognitive circuitry in Parkinson’s disease. Lancet Neurol..

[12] Spitzer NC (2017). Neurotransmitter switching in the developing and adult brain. Ann. Rev. Neurosci..

[13] Shiotsuki H (2010). A rotarod test for evaluation of motor skill learning. J. Neurosci. Methods.

[14] Buitrago MM (2004). Short and long-term motor skill learning in an accelerated rotarod training paradigm. Neurobiol. Learn. Mem..

[15] Pothakos K, Kurz MJ, Lau YS (2009). Restorative effect of endurance exercise on behavioral deficits in the chronic mouse model of Parkinson’s disease with severe neurodegeneration. BMC Neurosci..

[16] Mackintosh NJ (1975). A theory of attention: variations in the associability of stimuli with reinforcement. Psychological Rev..

[17] Morris RG (1986). Selective impairment of learning and blockade of long-term potentiation by an N-methyl-D-aspartate receptor antagonist, AP5. Nature.

[18] Marek KW, Kurtz LM, Spitzer NC (2010). cJun integrates calcium activity and tlx3 expression to regulate neurotransmitter specification. Nat. Neurosci..

[19] Güemez-Gamboa A (2014). Non-cell-autonomous mechanism of activity-dependent neurotransmitter switching. Neuron.

[20] Meng D (2018). Neuronal activity regulates neurotransmitter switching in the adult brain following light-induced stress. Proc. Natl Acad. Sci. USA.

[21] Clark PJ (2010). Adult hippocampal neurogenesis and c-Fos induction during escalation of voluntary wheel running in C57BL/6J mice. Behav. Brain Res..

[22] Boisgontier MP (2017). Individual differences in brainstem and basal ganglia structure predict postural control and balance loss in young and older adults. Neurobiol. Aging.

[23] Mena-Segovia J, Bolam JP (2017). Rethinking the pedunculopontine nucleus: from cellular organization to function. Neuron.

[24] Smith Y (2012). Parkinson’s disease therapeutics: new developments and challenges since the introduction of levodopa. Neuropsychopharmacol.

[25] Mena-Segovia J (2009). GABAergic neuron distribution in the pedunculopontine nucleus defines functional subterritories. J. Comp. Neurol..

[26] Tattersall TL (2014). Imagined gait modulates neuronal network dynamics in the human pedunculopontine nucleus. Nat. Neurosci..

[27] Thevathasan W (2012). Alpha oscillations in the pedunculopontine nucleus correlate with gait performance in parkinsonism. Brain.

[28] Gut NK, Winn P (2015). Deep brain stimulation of different pedunculopontine targets in a novel rodent model of parkinsonism. J. Neurosci..

[29] Karachi C (2010). Cholinergic mesencephalic neurons are involved in gait and postural disorders in Parkinson disease. J. Clin. Invest..

[30] Madisen L (2010). A robust and high-throughput Cre reporting and characterization system for the whole mouse brain. Nat. Neurosci..

[31] Chen E (2018). Altered baseline and nicotine-mediated behavioral and cholinergic profiles in ChAT-Cre mouse lines. J. Neurosci..

[32] Vincent SR (1983). NADPH-diaphorase: a selective histochemical marker for the cholinergic neurons of the pontine reticular formation. Neurosci. Lett..

[33] Saunders A, Granger AJ, Sabatini BL (2015). Corelease of acetylcholine and GABA from cholinergic forebrain neurons. elife.

[34] Wang HL, Morales M (2009). Pedunculopontine and laterodorsal tegmental nuclei contain distinct populations of cholinergic, glutamatergic and GABAergic neurons in the rat. Eur. J. Neurosci..

[35] Luquin E (2018). Stereological estimates of glutamatergic, GABAergic, and cholinergic neurons in the pedunculopontine and laterodorsal tegmental nuclei in the rat. Front. Neuroanat..

[36] Xiao C (2016). Cholinergic mesopontine signals govern locomotion and reward through dissociable midbrain pathways. Neuron.

[37] Dautan D (2016). Segregated cholinergic transmission modulates dopamine neurons integrated in distinct functional circuits. Nat. Neurosci..

[38] Gambhir H, Mathur R, Behari M (2011). Progressive impairment in motor skill learning at 12 and 20 weeks post 6-OHDA- SNc lesion in rats. Parkinsonism Relat. Disord..

[39] Leemburg. S (2018). Motor skill learning and reward consumption differentially affect VTA activation. Sci. Rep..

[40] Steriade M (1991). Fast oscillations (20-40 Hz) in thalamocortical systems and their potentiation by mesopontine cholinergic nuclei in the cat. Proc. Natl Acad. Sci. USA.

[41] Jeljeli M (2003). Effects of ventrolateral-ventromedial thalamic lesions on motor coordination and spatial orientation in rats. Neurosci. Res..

[42] Kaita AA, Goldberg AM (1969). Control of acetylcholine synthesis—the inhibition of choline acetyltransferase by acetylcholine. J. Neurochem..

[43] Morris D, Maneckjee A, Hebb C (1971). The kinetic properties of human placental choline acetyltransferase. Biochem. J..

[44] Dulcis D (2017). Neurotransmitter switching regulated by miRNAs controls changes in social preference. Neuron.

[45] Perez-Lloret S, Barrantes FJ (2016). Deficits in cholinergic neurotransmission and their clinical correlates in Parkinson’s disease, N.P.J. Parkinsons Dis..

[46] Hosp JA (2011). Dopaminergic projections from midbrain to primary motor cortex mediate motor skill learning. J. Neurosci..

[47] Guo L (2015). Dynamic rewiring of neural circuits in the motor cortex in mouse models of Parkinson’s disease. Nat. Neurosci..

[48] Vitrac C, Benoit-Marand M (2017). Monoaminergic modulation of motor cortex function. Front. Neural Circuits.

[49] Chandler DJ, Gao WJ, Waterhouse BD (2014). Heterogeneous organization of the locus coeruleus projections to prefrontal and motor cortices. Proc. Natl Acad. Sci. USA.

[50] MacLaren DA (2014). Deficits in motor performance after pedunculopontine lesions in rats – impairment depends on demands of task. Eur. J. Neurosci..

[51] Caggiano V (2018). Midbrain circuits that set locomotor speed and gait selection. Nature.

[52] Roseberry TK (2016). Cell-type-specific control of brainstem locomotor circuits by basal ganglia. Cell.

[53] Lee AM (2014). Identification of a brainstem circuit regulating visual cortical state in parallel with locomotion. Neuron.

[54] Mehrholz J (2015). Treadmill training for patients with Parkinson’s disease. Cochrane Database Syst. Rev..

[55] Polese JC (2013). Treadmill training is effective for ambulatory adults with stroke: a systematic review. J. Physiother..

[56] Gaßner H (2019). Perturbation treadmill training improves clinical characteristics of gait and balance in Parkinson’s Disease. J. Parkinsons Dis..

[57] Franklin K, Paxinos G (2008). The Mouse Brain In Stereotaxic Coordinates.

[58] Abs E (2018). Learning-related plasticity in dendrite-targeting layer 1 interneurons. Neuron.

[59] Knowland D (2017). Distinct ventral pallidal neural populations mediate separate symptoms of depression. Cell.

