# Supplementary material for: Exercise enhances motor skill learning by neurotransmitter switching in the adult midbrain
Source: Nat Commun. 2020 May 4;11:2195. doi: 10.1038/s41467-020-16053-7 (PMC7198516; doi:10.1038/s41467-020-16053-7)
Supplement: Supplementary file 1 — Supplementary Information [file 41467_2020_16053_MOESM1_ESM.pdf]

SUPPLEMENTARY INFORMATION

Exercise enhances motor skill learning by neurotransmitter switching in the adult midbrain

Hui-quan Li* and Nicholas C. Spitzer*

Nature Communications

Li & Spitzer, Supplementary Figure 1

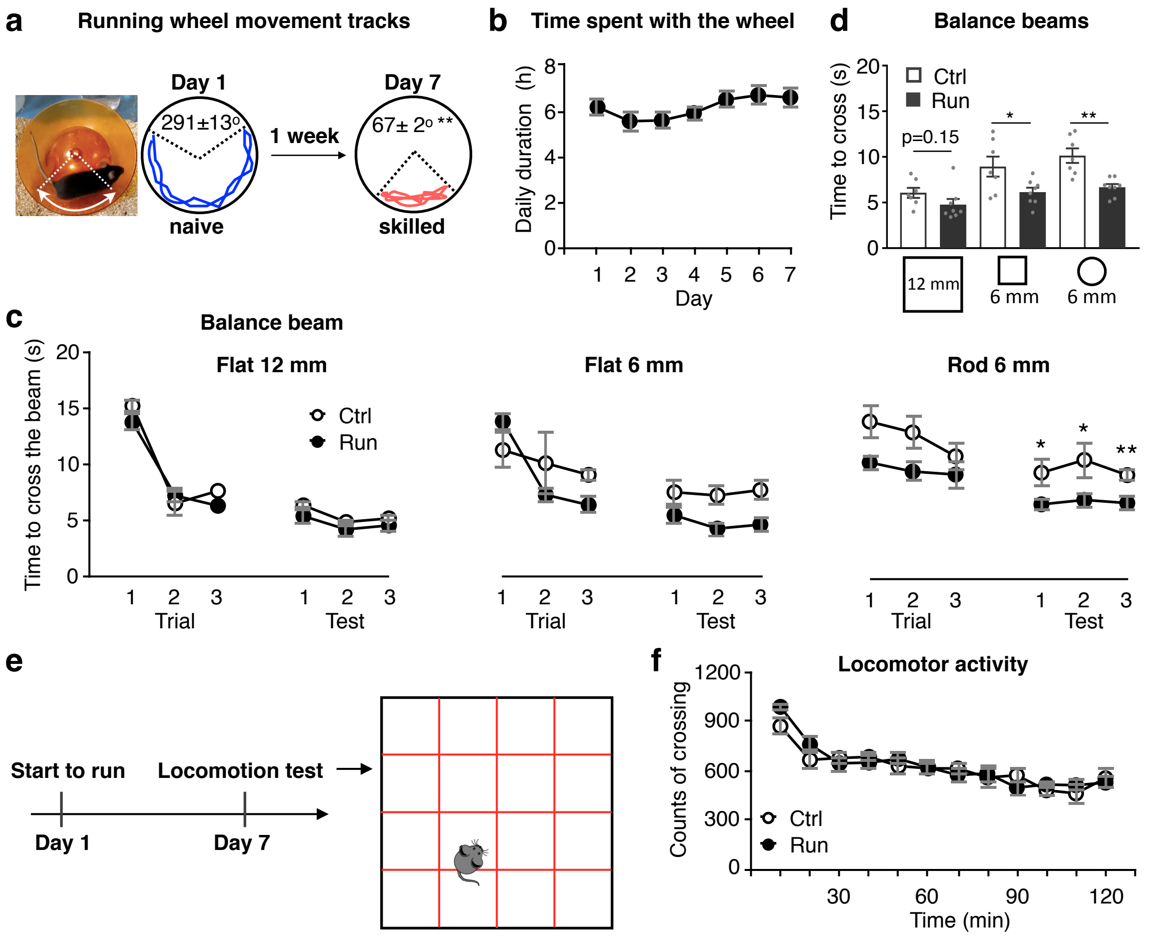

Supplementary Figure 1. Running enhances motor skill learning but does not affect basic locomotor activity, related to Figure 1. a Image of a runner mouse and examples of mouse movement tracks on the running wheel. Angles are mean mouse movements subtended while running. n=6 tracks from 3 animals per group. **b** Daily duration that mice spent with running wheels during one week of running. n=8 animals per group. **c** Time to cross a 1 meter long, 0.75 meter high balance beam during each trial of training or each test on the day after training. Beam shape (flat or rod) and size (diameter) are indicated. **d** Mean time to cross the balance beams in three tests on the day after training. For (**c**,**d**) n=7 animals for Ctrl and 8 animals for Run. **e** Experimental design for locomotion test. **f** Average counts of mice crossing laser beams in the novel locomotion chamber (**e**) in a 2-hour test. n=6 animals per group. Statistical significance *p<0.05, **p<0.01 was assessed by two-sided Welch's t-test. Data shown are mean± SEM.

Li & Spitzer, Supplementary Figure 2

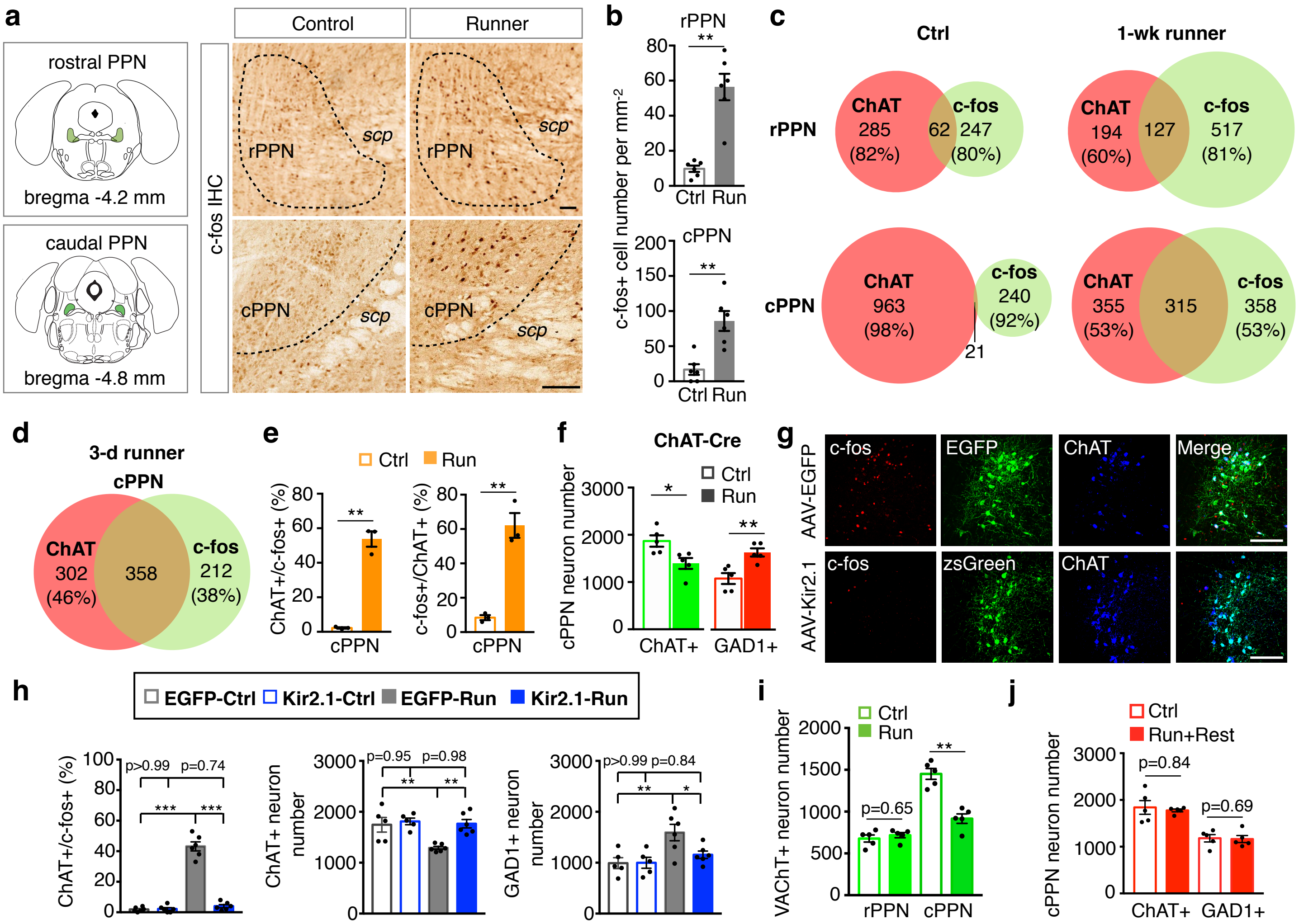

Supplementary Figure 2. Running activates cholinergic neurons and blocking the cholinergic cPPN activity prevents the changes in the numbers of ChAT and GAD1 neurons caused by running, related to Figures 2. a Left panels show the PPN (green) in coronal brain sections. The coronal brain sections were drawn according to the Franklin & Paxinos brain atlas (*57*). Middle and right panels illustrate 3,3'-diaminobenzidine (DAB) immunohistochemistry of c-fos in rostral PPNs (rPPN, upper) or caudal PPNs (cPPN, lower) of controls and 1-week runners. Dotted lines outline the PPN. Dark brown stain indicates c-fos+ neurons. Scale bar, 100 µm. **b** Counts of c-fos+ neurons in the rostral and caudal PPN. For (**a**,**b**), n=6 animals per group. **c** Co-localization of ChAT and c-fos in the PPN of controls and 1-wk runners. Cell numbers and percentages of cholinergic (red) and non-cholinergic (green) neurons are shown. n=3 animals per group. **d** Co-localization of ChAT and c-fos in the caudal PPN of mice that had run for 3 days. Cell numbers and percentages are shown. n=3 animals. **e** The percentage of the ChAT+, c-fos+ neurons in the ChAT+ neuron population (left) or in the c-fos+ neuron population (right) in the cPPN of 3-day runners and controls. n=3 animals per group. **f** Running for 1 week decreases the number of ChAT+ neurons and increases the number of GAD1+ neurons in the caudal PPN of a ChAT-IRES-Cre mouse line. n=5 animals per group. **g** Triple labeling of c-fos, EGFP or zsGreen and ChAT in 1-week runners that were injected with AAV-DIO-EGFP or AAV-DIO-Kir2.1-zsGreen. Scale bar, 100 µm. **h** Summary of (**g**) and non-runner controls. For (**g**,**h**), n=5 animals for Ctrl groups and 6 animals for Run groups. **i** Stereological counts of VAChT-immunostained neurons. n=5 animals per group. **j** Running-induced transmitter switching reverses after 1 week of rest, even though mice experienced behavioral tests immediately after running and retests one week later. n=5 animals per group. For (**f**,**h**,**j**), ChAT was detected by DAB staining. GAD1 was detected by *in situ* hybridization. Statistical significance **p*<0.05, ***p*<0.01, ****p*<0.001 was assessed by ANOVA (**h**) or Mann–Whitney U test (other panels). Data shown are mean± SEM.

Li & Spitzer, Supplementary Figure 3

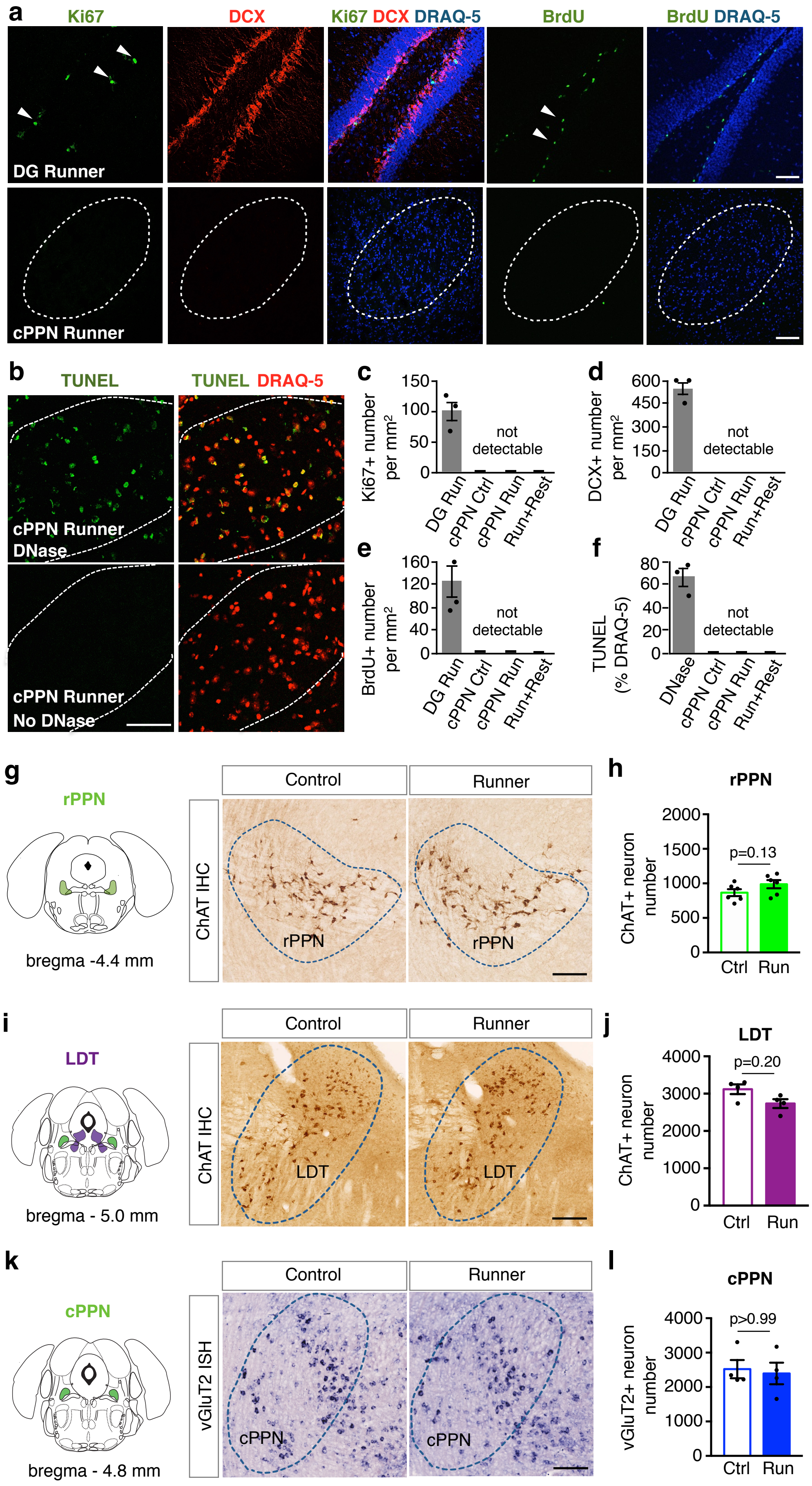

Li & Spitzer, Supplementary Figure 3

Supplementary Figure 3. No neurogenesis, apoptosis, change in the number of ChAT+ rPPN neurons or change in the number of vGluT2+ cPPN neurons was observed, related to Figure 2. a Sections of dentate gyrus (DG, upper panels) and cPPN (lower panels) from 1-week runners triple-stained for Ki67, DCX, and DRAQ-5 (nuclear marker) (Columns 1-3) or double-stained for BrdU and DRAQ-5 (columns 4-5). For BrdU labeling, mice were i.p. injected with BrdU (50 mg/kg) once every 12 hr for 1 week. Dotted lines outline the cPPN. Arrowheads point to Ki67+ (upper panel in column 1) or BrdU+ cells (upper panel in column 4). Scale bar, 100 µm. **b** Sections of the cPPN of a 1-wk runner are double-stained for TUNEL and DRAQ-5. Dotted lines outline the cPPN. Upper panels: DNase-treated tissue as positive control. Lower panels: no DNase treatment. Scale bar, 100 µm. **c-e** Summary of (**a**) combined with littermate non-runner controls and mice that ran for 1 week followed by 1 week of rest. DG Run, dentate gyrus of a runner mouse. The region of interest that was quantified includes only the granule layer and not the hilus of the dentate gyrus. For (**a**-**f**), n=3 animals, 12 sections per group. **f** Summary of (**b**) combined with littermate non-runner controls and mice that ran 1-week followed by 1 week of rest. n=3 animals, 12 sections per group. **g** The left panel shows the rPPN (green) in a coronal brain section. Middle and right panels illustrate DAB staining of ChAT in rPPN of a control and 1-week runner. Dotted lines outline the rPPN. Dark brown stain indicates ChAT+ neurons. Scale bar, 100 µm. **h** Stereological counts of ChAT+ neurons in the rostral PPN. For (**g**,**h**), n=6 animals per group. **i** The left panel shows the LDT (purple) and the caudal PPN (green) in coronal brain sections. Middle and right panels illustrate DAB staining of ChAT in LDT of a control and 1-week runner. Dotted lines outline the LDT. Dark brown stain indicates ChAT+ neurons. Scale bar, 100 µm. **j** Stereological counts of ChAT+ neurons in the LDT. For (**i**,**j**), n=4 animals per group. **k** The left panel shows the cPPN (green) in a coronal brain section. Middle and right panels illustrate *in situ* hybridization staining of vGluT2 of a control and a 1-week runner. Dotted lines outline the cPPN. Dark blue-purple stain indicates vGluT2+ neurons. Scale bar, 100 µm. **l** Stereological counts of vGluT2+ neurons in the cPPN. For (**k**,**l**), n=4 animals per group. The coronal brain sections in (**g**,**i**,**k**) were drawn according to the Franklin & Paxinos brain atlas (*57*). Statistical significance was assessed by Mann–Whitney U test. Data shown are mean± SEM.

Li & Spitzer, Supplementary Figure 4

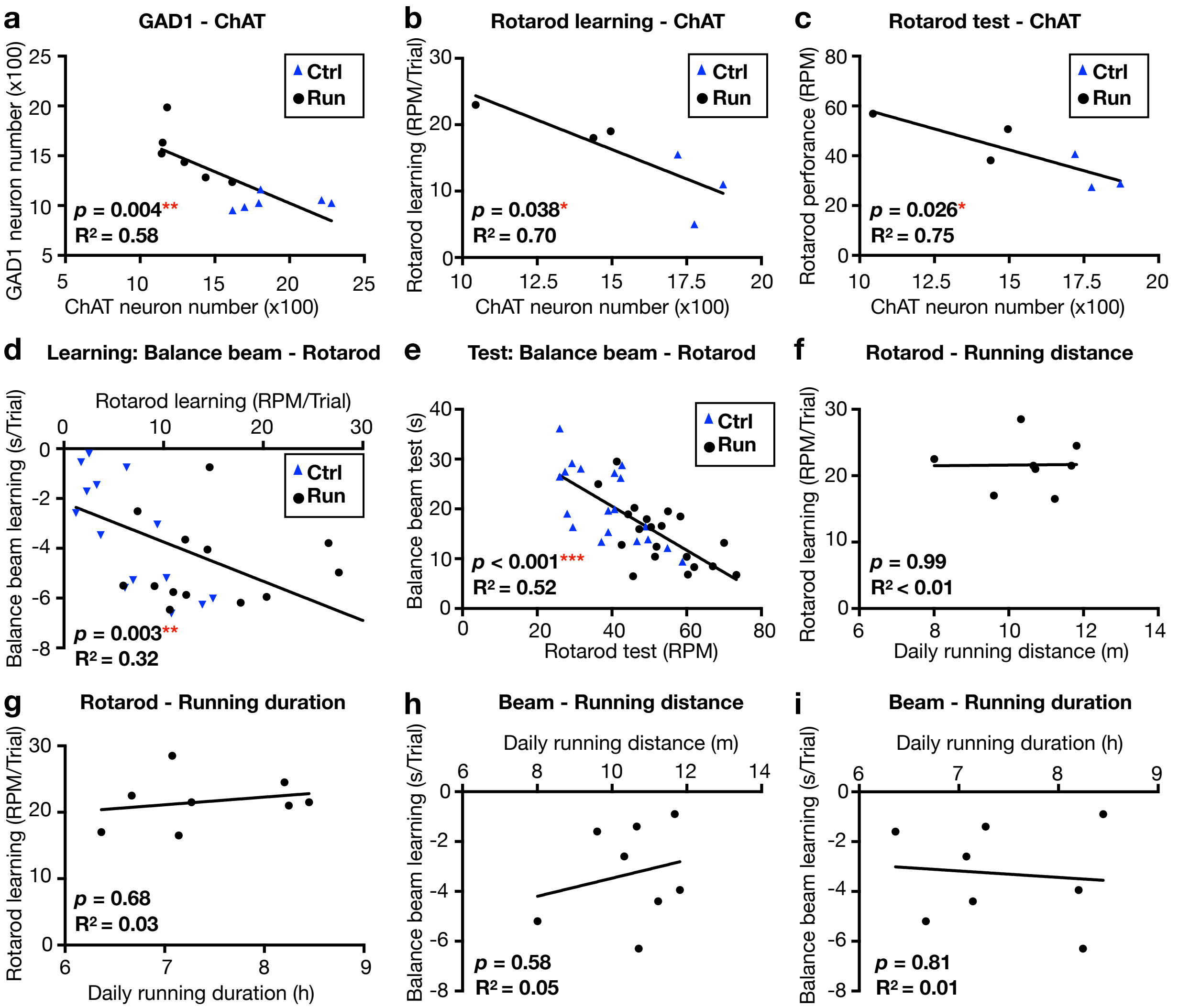

Supplementary Figure 4. Correlations of the extent of transmitter switching, motor skill learning, and wheel running, related to Figures 1 and 2. Linear regression and Pearson's correlation analysis were performed to analyze the correlation (**a**) between the number of GAD1+ neurons and the number of ChAT+ neurons in the cPPN, (**b**) between the learning slope of the rotarod training and the number of ChAT+ neurons in the cPPN, (**c**) between the performance of the rotarod test and the number of ChAT+ neurons in the cPPN, (**d**) between the learning slope of the balance beam training and the rotarod training, (**e**) between the performance of the balance beam test and the rotarod test, (**f**) between the learning slope of the rotarod training and the average daily running distance or (**g**) average daily running duration, (**h**) between the learning slope of the balance beam training and the average daily running distance or (**i**) average daily running duration. Each dot represents one animal. Ctrl, mice that had not run on a running wheel. Run, mice that had run for 1 week. For rotarod and balance beam, the learning slopes during training and the performance during test were determined as described in Figure 1. The trend lines, *p*-values and correlation coefficient values (R^2) are shown. *, p<0.05; **, p<0.01; ***, p<0.001.

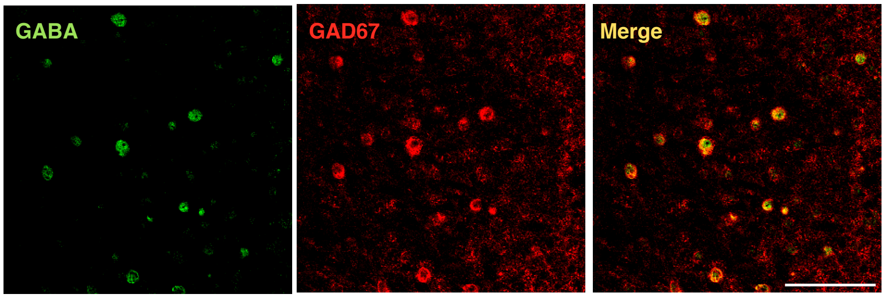

Supplementary Figure 5. Validation of the staining specificity of the anti-GABA antibody, related to Figure 3. Double staining of GABA and GAD67 in the superior colliculus of a wild-type mouse. More than 98% GABA+ neurons (243/246 cells quantified from 3 animals) are GAD67+. Scale bar, 100 µm.

Li & Spitzer, Supplementary Figure 6

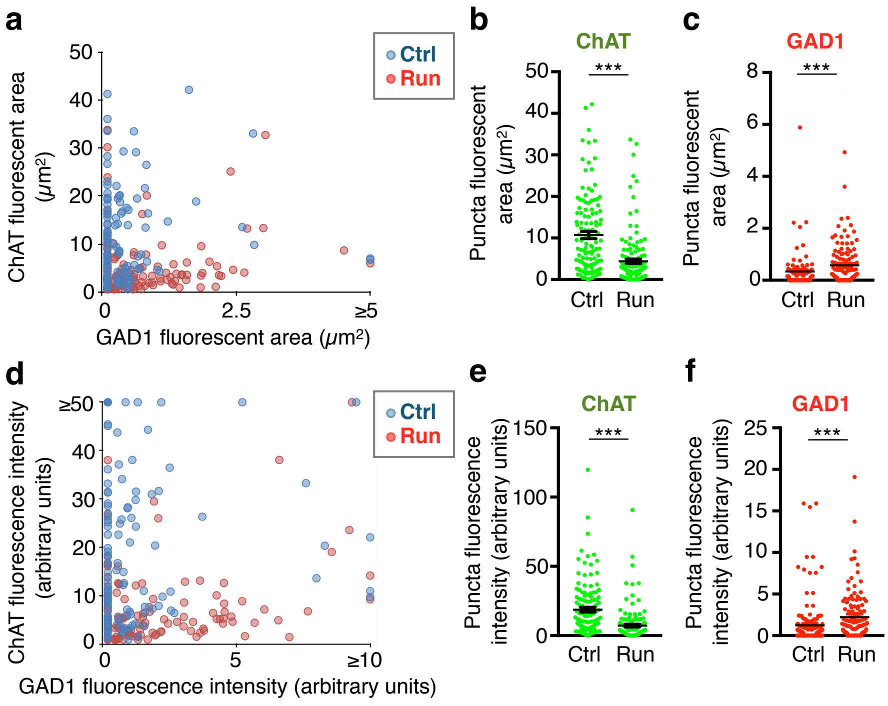

Supplementary Figure 6. Changes of ChAT and GAD1 transcript area and intensity in nNOS neurons, related to Figure 4. a Scatterplot of fluorescent area of ChAT transcripts (y-axis) against fluorescent area of GAD1 transcripts (x-axis) in nNOS neurons. Each dot represents one neuron. **b,c** Mean fluorescent area of ChAT (**b**) or GAD1 (**c**) transcripts in single nNOS+ cells. **d** Scatterplot of fluorescence intensity of ChAT transcripts (y-axis) against fluorescence intensity of GAD1 transcripts (x-axis) in nNOS cells. Each dot represents one neuron. **e,f** Mean fluorescence intensity of ChAT (**e**) or GAD1 (**f**) transcripts in single nNOS+ neurons. For (**a**-**f**), n=4 animals per group; n=123 cells for Ctrl and 137 cells for Run. Statistical significance ***p<0.001 was assessed by two-sided Welch's t-test. Data shown are mean± SEM.

Li & Spitzer, Supplementary Figure 7

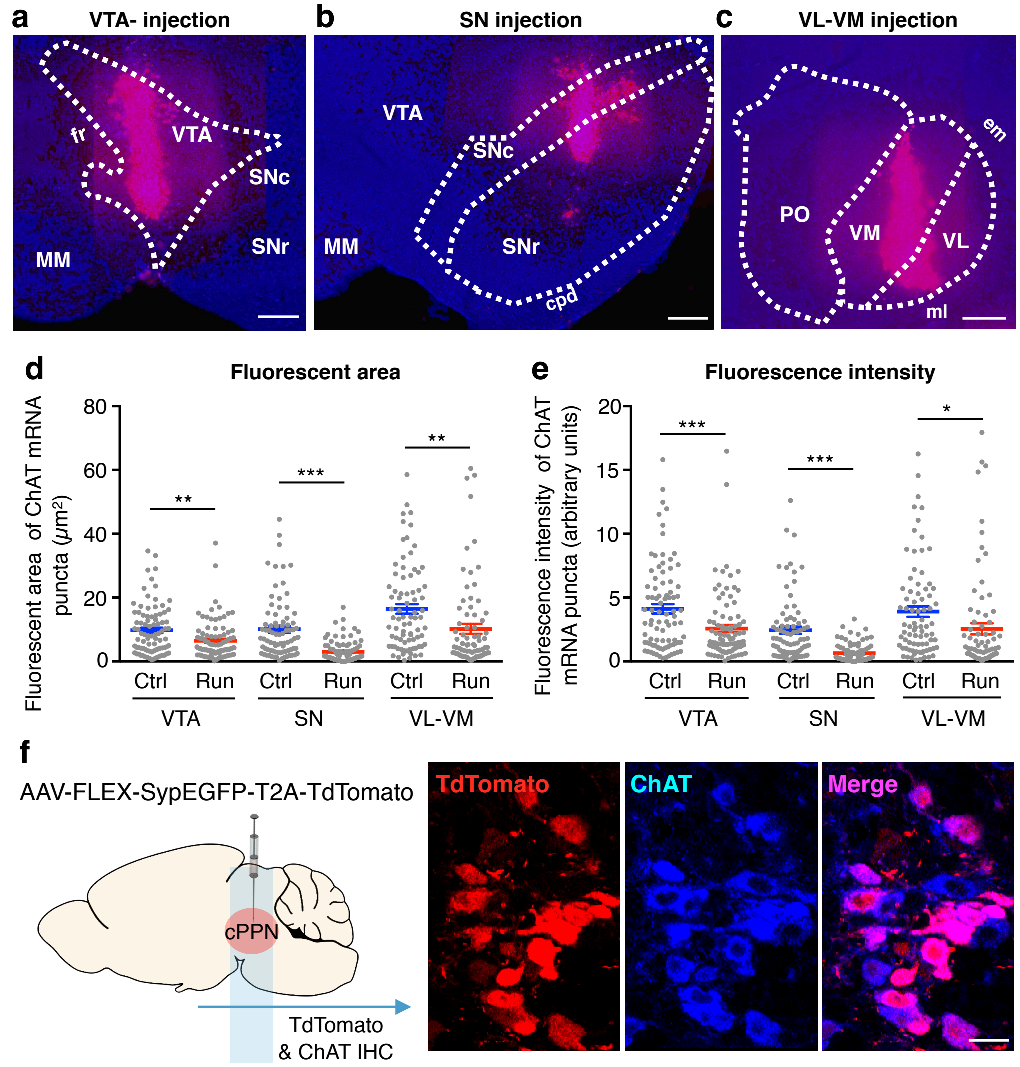

Supplementary Figure 7. Anatomical evidence of retrobead injection and fluorescent area/intensity of ChAT transcripts in nNOS neurons that project to each brain region, and validation of AAV-FLEX-SypEGFP-T2A-TdTomato, related to Figure 5. a-c Representative coronal sections show retrobeads (magenta) injected into the VTA (**a**), SN (**b**) and VL-VM (**c**). n=4 animals per brain region. MM, medial mammillary nucleus; fr, fasciculus retroflexus; SNc, substantia nigra, compact part; SNr, substantia nigra, reticular part; cpd, cerebral peduncle; PO, posterior complex of the thalamus; ml, medial lemniscus; em, external medullary lamina of the thalamus. The boundaries of the nuclei are drawn based on the comparison with the Allen mouse brain atlas. Scale bar, 200 μm. **d,e** Mean fluorescent area (**d**) or fluorescence intensity (**e**) of ChAT transcripts in nNOS neurons that project to corresponding brain regions (x-axis). Each dot represents one cell. n=4 animals per group. n=89 cells for VTA-Ctrl, 91 for VTA-Run, 81 for SN-Ctrl, 87 for SN-Run, 82 for VL-VM-Ctrl, 80 for VL-VM-Run. **f** Validation of the cell specificity of the AAV-FLEX-SypEGFP-T2A-TdTomato construct. The construct was injected into the cPPN of ChAT-Cre mice. Staining of the cPPN reveals co-localization of TdTomato and ChAT. n=6 animals. Scale bar, 20 μm. Statistical significance *p<0.05,**p<0.01, ***p<0.001 was assessed by two-sided Welch's t-test. Data shown are mean± SEM.

Li & Spitzer, Supplementary Figure 8

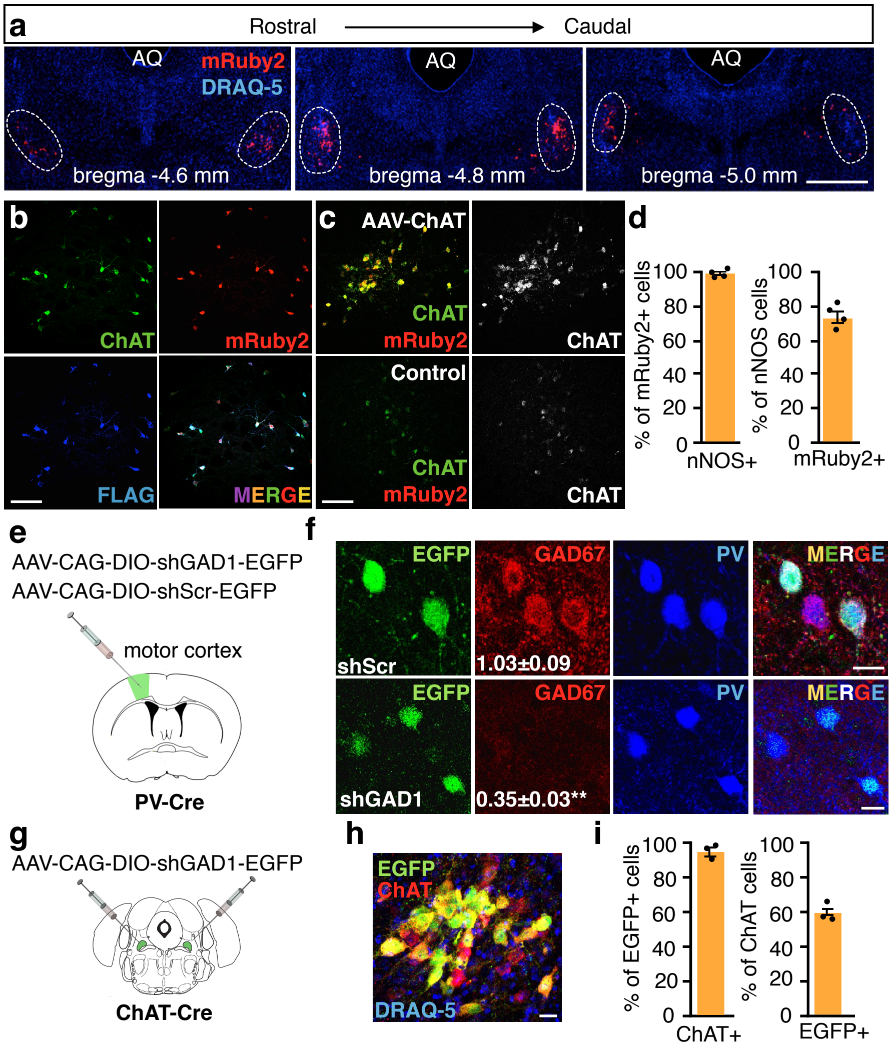

Supplementary Figure 8. Validation of the use of the AAV-DIO-ChAT and AAV-DIO-shGAD1 constructs, related to Figures 6 and 7. a mRuby2 expression in rostral-to-caudal sections of the cPPNs (dotted lines) of a single animal bilaterally-injected with AAV-hSyn-DIO-ChAT-flag-P2A-mRuby2 (AAV-DIO-ChAT). Coordinates adapted to Allen Brain Atlas. AQ, aqueduct. Scale bar, 1 mm. n=34 animals. **b** Triple-labeled images of ChAT, mRuby2, FLAG and merged image in a cPPN of an animal injected with AAV-DIO-ChAT. Scale bar, 100 µm. n=3 animals. **c** Double-stained images of ChAT and FLAG in both AAV-DIO-ChAT-injected (upper panels) or contralateral uninjected control (lower panels). Statistical analysis is in Figure 5c. Scale bar, 100 µm. n=3 animals per group. **d** The percentage of nNOS+mRuby2+ neurons in the mRuby2+ population (cell type specificity) and the percentage of nNOS+mRuby2+ population in the nNOS+ neurons (transfection efficiency). n=4 animals per group. **e** Knockdown efficiency of GAD67 was verified *in vivo* by expressing either AAV-CAG-DIO-GAD1-shRNA-EGFP (AAV-DIO-shGAD1) or AAV-DIO-scramble-shRNA-EGFP (AAV-DIO-shScr) constructs in the cortex of a PV-Cre mouse. **f** Brain sections from (**e**) were immunostained for GAD67 and PV**.** Scale bar, 20 µm. The GAD67+ intensities of EGFP+ neurons relative to non-transfected PV+ cells are shown. n=78 cells for AAV-DIO-shScr and 107 cells for AAV-DIO-shGAD1, from 3 animals per group. **g,h** Triple-labeled images of EGFP, ChAT, and DRAQ-5 in the cPPN of a non-runner ChAT-IRES-Cre mouse injected with AAV-DIO-shGAD1. **i** The specificity of transduction was measured as the percentage of ChAT+EGFP+ neurons in the EGFP population (>95%, 197/208) and the efficiency was measured as the percentage of ChAT+EGFP+ neurons in the ChAT population (~60%, 197/330). Similar specificity (>95%) and efficiency (~60%) were observed for the control shScr construct. The coronal brain sections in (**e**,**g**) were drawn according to the Franklin & Paxinos brain atlas (*57*). For (**h**,**i**), n=3 animals per group. Data shown are mean±SEM.

Li & Spitzer, Supplementary Figure 9

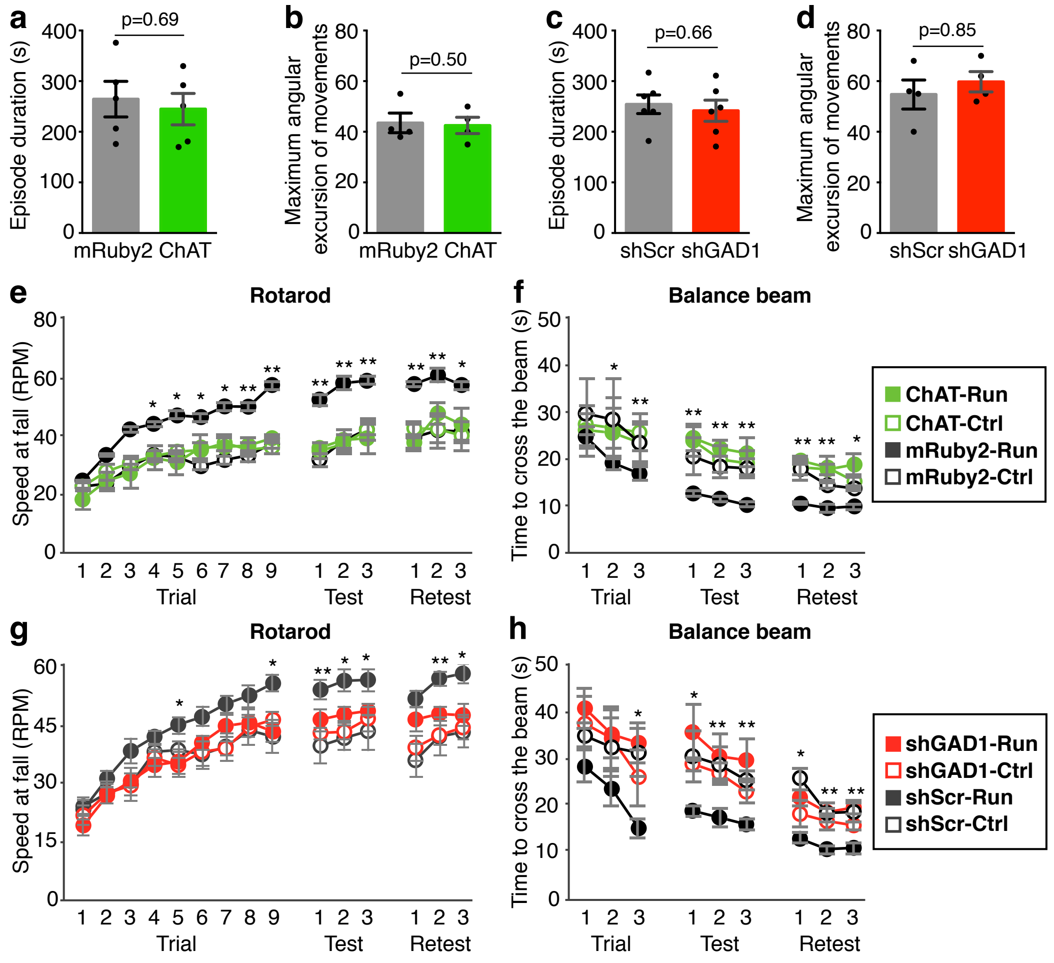

Supplementary Figure 9. Running is not affected but running-Induced improvements in motor skill learning are impaired when transmitter switching is overridden, related to Figures 6 and 7. a-d Mean duration of running episodes and maximum angular excursion of mouse movements on the running wheels for 1-week trained ChAT-Cre runner mice that were injected with AAV-DIO-mRuby2, AAV-DIO-ChAT, AAV-DIO-shScr, or AAV-DIO-shGAD1 in the cPPN. **a** n=5 animals per each group. **c** n=6 animals for each group. **b,d** n=4 animals per each group. **e,f** Mean speed at fall on a rotarod and mean time to cross a 1 meter long, 0.75 meter high, 4 mm rod balance beam at each trial during training, at each test on the day after training, and at each retest 1 week after test. n=9 animals for mRuby2-Run and 18 animals for ChAT-Run. **g,h** Same as (**e,f**). n=8 animals for shScr-Run and 9 for shGAD1-Run. Statistical significance **p*<0.05, ***p*<0.01 was assessed by two-sided Welch's t-test. Data shown are mean± SEM.

Li & Spitzer, Supplementary Figure 10

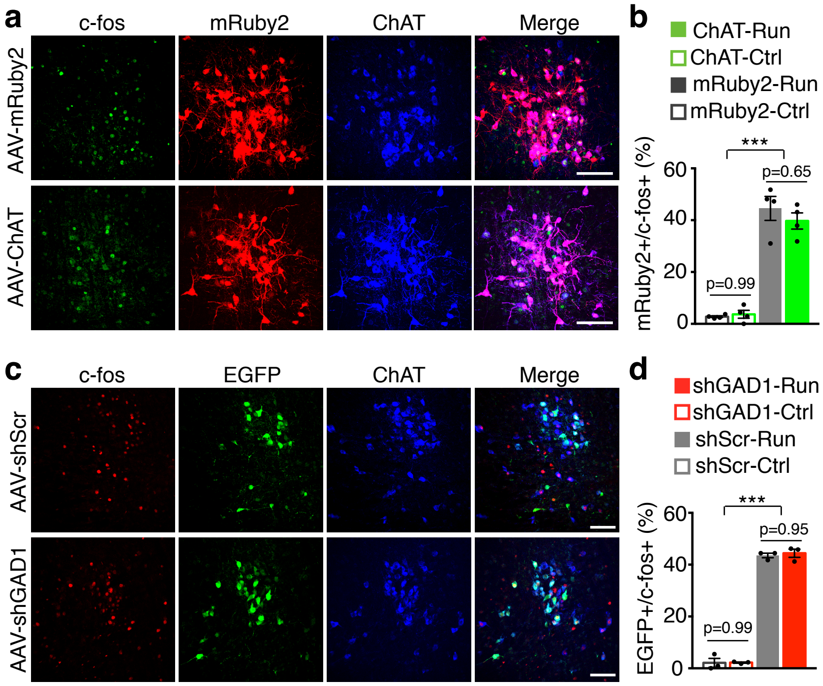

Supplementary Figure 10. Overriding transmitter switching does not affect c-fos expression in cholinergic cPPN neurons, related to Figures 6 and 7. a Triple labeling of c-fos, mRuby2 and ChAT in 1-week runners that were injected with AAV-DIO-mRuby2 or AAV-DIO-mRuby2-P2A-ChAT. Scale bar, 100 µm. **b** Summary of (**a**) and non-runner controls. n=214 cells for mRuby2-Ctrl, 204 for ChAT-Ctrl, 223 for mRuby2-Run and 206 for ChAT-Run. For (**a**,**b**), n=4 animals per group. **c** Triple labeling of c-fos, EGFP and ChAT in 1-week runners that were injected with AAV-DIO-shScr-EGFP or AAV-DIO-shGAD1-EGFP. Scale bar, 100 µm. **d** Summary of (**c**) and non-runner controls. n=213 cells for shScr-Ctrl, 360 for shGAD1-Ctrl, 277 for shScr-Run and 315 for shGAD1-Run. For (**c**,**d**), n=3 animals per group. Statistical significance ***p<0.001 was assessed by ANOVA followed by Tukey's test. Data shown are mean± SEM.
