# Supplementary material for: Exercise enhances motor skill learning by neurotransmitter switching in the adult midbrain
Source: Nat Commun. 2020 May 4;11:2195. doi: 10.1038/s41467-020-16053-7 (PMC7198516; doi:10.1038/s41467-020-16053-7)
Supplement: Supplementary file 2 — Reporting Summary [file 41467_2020_16053_MOESM2_ESM.pdf]

nature research

Corresponding author(s): Hui-quan Li, Nicholas C. Spitzer

Last updated by author(s): 2020/03/24

Reporting Summary

Nature Research wishes to improve the reproducibility of the work that we publish. This form provides structure for consistency and transparency in reporting. For further information on Nature Research policies, see Authors & Referees and the Editorial Policy Checklist .

Statistics

For all statistical analyses, confirm that the following items are present in the figure legend, table legend, main text, or Methods section.

n/a	Confirmed	
☐	☒	The exact sample size (*n*) for each experimental group/condition, given as a discrete number and unit of measurement
☐	☒	A statement on whether measurements were taken from distinct samples or whether the same sample was measured repeatedly
☐	☒	The statistical test(s) used AND whether they are one- or two-sided<br>*Only common tests should be described solely by name; describe more complex techniques in the Methods section.*
☐	☒	A description of all covariates tested
☐	☒	A description of any assumptions or corrections, such as tests of normality and adjustment for multiple comparisons
☐	☒	A full description of the statistical parameters including central tendency (e.g. means) or other basic estimates (e.g. regression coefficient) AND variation (e.g. standard deviation) or associated estimates of uncertainty (e.g. confidence intervals)
☐	☒	For null hypothesis testing, the test statistic (e.g. *F*, *t*, *r*) with confidence intervals, effect sizes, degrees of freedom and *P* value noted<br>*Give P values as exact values whenever suitable.*
☒	☐	For Bayesian analysis, information on the choice of priors and Markov chain Monte Carlo settings
☒	☐	For hierarchical and complex designs, identification of the appropriate level for tests and full reporting of outcomes
☐	☒	Estimates of effect sizes (e.g. Cohen's *d*, Pearson's *r*), indicating how they were calculated

Our web collection on statistics for biologists contains articles on many of the points above.

Software and code

Policy information about availability of computer code

Data collection	JWatcher version 1.0, Stereo Investigator version 11.08. 1.64-bit
Data analysis	ImageJ - fiji 1.52P, GraphPad Prism version 7, MATLAB R2018b, Leica Application Suite X

For manuscripts utilizing custom algorithms or software that are central to the research but not yet described in published literature, software must be made available to editors/reviewers. We strongly encourage code deposition in a community repository (e.g. GitHub). See the Nature Research guidelines for submitting code & software for further information.

Data

Policy information about availability of data

All manuscripts must include a data availability statement. This statement should provide the following information, where applicable:

- Accession codes, unique identifiers, or web links for publicly available datasets
- A list of figures that have associated raw data
- A description of any restrictions on data availability

All data generated or analyzed during this study are included in this published article and its supplementary information files. Source data are provided as a Source Data file.

Field-specific reporting

Please select the one below that is the best fit for your research. If you are not sure, read the appropriate sections before making your selection.

☒ Life sciences ☐ Behavioural & social sciences ☐ Ecological, evolutionary & environmental sciences

Life sciences study design

All studies must disclose on these points even when the disclosure is negative.

Sample size	Number of cells and number of animals are on a par with the following papers in the field. Esposito, M.S. et al. Brainstem nucleus MdV mediates skilled forelimb motor tasks. Nature 508, 351-356 (2014). Caggiano, V. et al. Midbrain circuits that set locomotor speed and gait selection. Nature 553, 455-460 (2018). Capelli .P. et al. Locomotor speed control circuits in the caudal brainstem. Nature 551:373-377 (2017).
Data exclusions	No data were excluded in this study.
Replication	Experiments were carried out independently twice (Figs. 2b,c, 4a,b, 5b,c, h-j, 7b and Supplementary Figs. 1f, 3a-f, 5, 7f, 8f), three times (Figs. 1b, h-j, 2d-k, 3, 4c-h, 5e-f, 6c-d, 7c, g, and Supplementary Figs. 1a-d, 2b-f, i-j, 3g-l, 4a-c, f-i, 6, 7d-e, 8, 9a-d and 10), or four or more times (Figs. 1c-f, 6e-g, 7d-f and Supplementary Figs. 2g,i, 4d,e, 9e-h). All attempts at replication were successful.
Randomization	The assignment of animals to control or experimental groups was randomized.
Blinding	Investigators were double-blinded to the history of the mice when doing behavior analysis that required hand scoring or performing manual cell counting.

Reporting for specific materials, systems and methods

We require information from authors about some types of materials, experimental systems and methods used in many studies. Here, indicate whether each material, system or method listed is relevant to your study. If you are not sure if a list item applies to your research, read the appropriate section before selecting a response.

Materials & experimental systems

n/a	Involved in the study
☐	☒ Antibodies
☒	☐ Eukaryotic cell lines
☒	☐ Palaeontology
☐	☒ Animals and other organisms
☒	☐ Human research participants
☒	☐ Clinical data

Methods

n/a	Involved in the study
☒	☐ ChIP-seq
☒	☐ Flow cytometry
☒	☐ MRI-based neuroimaging

Antibodies

Antibodies used	Primary antibodies used in this study were goat anti-ChAT (Millipore, AB144P), rabbit-anti-nNOS (Thermo Fisher, 61-7000), goat anti-cFos (Santa Cruz, sc-52G), rabbit-anti-cFos (Santa Cruz, sc-52), mouse-anti-cFos (Abcam, ab208942), rabbit-anti-PV (Swant, PV25), goat-anti-VAChT (Millipore, ABN100), goat-anti-VGAT (Synaptic system, 131004, 1:1000), mouse-anti-NeuN (Millipore, MAB377), rabbit-anti-GABA (Sigma-Aldrich, A2052) rabbit-anti-GFP (Thermo Fisher, A11122), chicken anti-GFP (Abcam, ab13970), guinea pig anti-GFP (Synaptic Systems,132005), rabbit-anti-zsGreen (Takara, 632474, 1:500), goat-anti-doublecortin (Santa Cruz, sc-8066), rabbit-anti-Ki67 (Cell Signaling, 9129) and rat-anti BrdU antibody (AbD Serotec, MCA2060). Secondary antibodies for immunofluorescence were from Jackson ImmunoResearch Labs: Alexa Fluor-488 donkey-anti-rabbit (705-545-003), Alexa Fluor-488 donkey-anti-guinea pig (706-545-148), Alexa Fluor-488 donkey-anti-mouse (715-545-150), Alexa Fluor-488 donkey-anti-goat (705-545-147), Alexa Fluor-594 donkey-anti-goat (705-585-147), Alexa Fluor-594 donkey-anti-mouse (715-585-150), and Alexa Fluor-647 donkey-anti-goat (705-605-147). Alexa Fluor-647 donkey-anti-rabbit (711-605-152). Alexa Fluor-488 donkey anti-rat antibody (A21208) was from Life Technologies. Biotinylated goat anti-rabbit (BA-1000) and horse anti-goat (BA-9500) secondary antibodies for DAB staining were from Vector Laboratories. Anti-Digoxigenin-AP Fab fragments anbibody (11093274910) was from Roche.
Validation	Goat anti-ChAT antibody (Millipore, AB144P) has been published and validated for use in immunohistochemistry (paraffin), immunocytochemistry, immunohistochemistry (frozen) and Western Blot. It has been validated to react with human, rat, mouse, monkey, opossum, avian, chick, guinea pig and zebrafish.<br><br>Rabbit-anti-nNOS antibody (Thermo Fisher, 61-7000) has been published and validated for use in ELISA, immunohistochemistry (frozen), immunohistochemistry (paraffin), immunofluorescence, Western Blot, immunofluorescence, immunohistochemistry (free floating, immunomicroscopy, Miscellaneous pubMed, immunocytochemistry, and immunoprecipitation. It has been validated to react with human, rat, mouse.<br><br>Goat anti-cFos antibody (Santa Cruz, sc-52G) has been validated for detection of cFos by Western Blot (starting dilution 1:200, dilution range 1:100- 1:1000), immunoprecipitation [1–2 µg per 100–500 µg of total protein (1 ml of cell lysate)], immunofluorescence and immunohistochemistry (including paraffin-embedded sections) (starting dilution 1:50, dilution range 1:50- 1:500) and flow cytometry (1 µg per 1 x 106 cells). It has been validated to react with mouse, rat and human.

Rabbit-anti-cFos antibody (Santa Cruz, sc-52) is discontinued and replaced by sc-166940. It is recommended for detection of c-Fos, Fos B, Fra-1 and Fra-2 by Western Blot, immunoprecipitation, immunofluorescence, immunohistochemistry (paraffin) and ELISA. It is reactive with mouse, rat, human, zebrafish, equine, canine, bovine, porcine and avian.

Mouse-anti-cFos antibody (Abcam, ab208942) has been published and validated for use in immunohistochemistry (frozen), immunohistochemistry (paraffin), immunofluorescence, Western Blot, immunofluorescence, immunohistochemistry (free Floating). It has been validated to react with human, rat, mouse.

Rabbit-anti-PV antiserum (Swant, PV25) is discontinued and replaced by PV27. It has been published and validated for use in immunohistochemistry with the avidin-biotin method and immunoblots. It has been validated to react with human, rat, mouse.

Goat-anti-VAChT antibody (Millipore, ABN100) has been published and validated for use in ELISA and immunohistochemistry. It has been validated to react with rat and mouse.

Goat-anti-VGAT antibody (Synaptic system, 131004, 1:1000) has been published and validated for use in immunohistochemistry (paraffin), immunocytochemistry, immunohistochemistry (frozen), Western Blot and immunoprecipitation. It has been validated to react with rat, mouse, zebrafish and ape.

Mouse-anti-NeuN antibody (Millipore, MAB377) has been published and validated for use in flow cytometry, immunohistochemistry (paraffin), immunocytochemistry, immunohistochemistry (frozen), Western Blot and immunoprecipitation. It has been validated to react with avian, chicken, ferret, human, mouse, pig, rat and salamander.

Rabbit-anti-GABA antibody (Sigma-Aldrich, A2052) has been published and validated for use in immunohistochemistry (paraffin) and dot blot. It has been validated to react with a wide range of species and has been published for use in rat and Drosophila.

Rabbit-anti-GFP antibody (Thermo Fisher, A11122) has been published and validated for use in ELISA, immunohistochemistry (frozen), immunohistochemistry (paraffin), immunofluorescence, Western Blot, immunohistochemistry (free floating), immunomicroscopy, miscellaneous pubMed, immunocytochemistry, ChIP assay, flow cytometry, gel shift, in situ hybridization, neutralization and immunoprecipitation. It has been published and validated for use in chimpanzee, dog, fish, Drosophila, hamster, human, mouse, non-human primate, pig, rat, sheep, tag, zebrafish.

Chicken anti-GFP antibody (Abcam, ab13970) has been published and validated for use in immunohistochemistry (wholemount), immunohistochemistry (paraffin), immunofluorescence, Western Blot, immunocytochemistry, immunohistochemistry (free floating) and immunohistochemistry (frozen).

Guinea pig anti-GFP antibody (Synaptic Systems,132005) has been validated for use in immunohistochemistry (paraffin), Western Blot, immunocytochemistry, immunohistochemistry (frozen) and immunoprecipitation.

Rabbit-anti-zsGreen antibody (Takara, 632474) has been validated for Western blotting and immunoprecipitation.

Goat-anti-doublecortin antibody (Santa Cruz, sc-8066) has been published and validated for use in Western Blot, immunocytochemistry, Miscellaneous PubMed and immunofluorescence. It has been validated to react with human, mouse and non-human primate.

Rabbit-anti-Ki67 antibody (Cell Signaling, 9129) has been published and validated for use in flow cytometry, immunocytochemistry and immunofluorescence (frozen). It has been validated for use in human, mouse and rat.

Rat-anti BrdU antibody (AbD Serotec, MCA2060) has been validated for use in immunohistochemistry, immunocytochemistry, flow cytometry, immunohistochemistry (paraffin), immunohistochemistry (frozen) and immunohistochemistry (free floating). It has been validated and published for use in human, mouse and rat.

Animals and other organisms

Policy information about studies involving animals; ARRIVE guidelines recommended for reporting animal research

Laboratory animals: C57BL/6J (JAX#000664),ChAT-IRES-Cre (JAX#006410) and PV-IRES-C re (JAX#008069) mice were used in the study. All expenments were performed on 8- to 12-week-old male mice. Mice were maintained on a 12 h:12 h light:dark cycle (light on: 10:00 pm-10:00 am) with food and water ad libitum. Vivarium temperature was between 65-75°F (~18-23°C) with 40-60% humidity.

Wild animals: No wild animals were used in this study.

Field-collected samples: No field-collected samples were used in this study.

Ethics oversight: All animal procedures were carried out in accordance with NIH guidelines and approved by the University of California, San Diego Institutional Animal Care and Use Committee or Scripps Institutional Animal Care and Use Committee.

Note that full information on the approval of the study protocol must also be provided in the manuscript.
